# A targetable antioxidant defense mechanism to EZH2 inhibitors enhances tumor cell vulnerability to ferroptosis

**DOI:** 10.1038/s41419-025-07607-y

**Published:** 2025-04-14

**Authors:** Marta C. Nocito, Constanze Hantel, Antonio M. Lerario, Fabrizio Mastrorocco, Luca De Martino, Clara Musicco, Ida D. Perrotta, Mariafrancesca Scalise, Cesare Indiveri, Sergio Giannattasio, Pierre Val, Marilena Lanzino, Vincenzo Pezzi, Ivan Casaburi, Rosa Sirianni

**Affiliations:** 1 Department of Pharmacy and Health and Nutritional Sciences, University of Calabria, 87036 Rende, Italy; 2 Department of Endocrinology, Diabetology and Clinical Nutrition, University Hospital Zurich (USZ) and University of Zurich (UZH), 8091, Zürich, Switzerland; 3 Medizinische Klinik und Poliklinik III, University Hospital Carl Gustav Carus Dresden, 01307 Dresden, Germany; 4 Departments of Molecular and Integrative Physiology and Internal Medicine, University of Michigan, Medical School, 48104 Ann Arbor, MI USA; 5 Institute of Biomembranes, Bioenergetics and Molecular Biotechnologies (IBIOM), National Research Council of Italy (CNR), 70126 Bari, Italy; 6 Centre for Microscopy and Microanalysis (CM2), Department of Biology, Biology, Ecology and Earth Sciences (DiBEST), University of Calabria, 87036 Rende, Italy; 7 Department of Biology, Ecology and Earth Sciences (DiBEST), University of Calabria, 87036 Rende, Italy; 8 France iGReD (Institute of Genetics, Reproduction and Development), CNRS UMR 6293, Inserm U1103, Université Clermont Auvergne, 28 Place Henri Dunant, 63000 Clermont-Ferrand, France; 9 Centro Sanitario, University of Calabria, Ponte P. Bucci, 87036 Rende, Italy

**Keywords:** Cancer metabolism, Targeted therapies

## Abstract

Epigenetic changes are present in all human cancers and are responsible for switching on or off genes, thus controlling tumor cell transcriptome. These changes occur through DNA methylation, histone modifiers and readers, chromatin remodelers, and microRNAs. The histone H3 methyl-transferase EZH2 gene is overexpressed in several cancer types, including adrenocortical carcinoma (ACC), a rare cancer still lacking a targeted therapy. EZH2 inhibitors (EZH2i) have been tested in several clinical trials, but their effectiveness was limited by the toxic effects of the therapeutic doses. We tested several EZH2i on ACC cells, and observed a significant reduction in cell growth only with doses much higher than those required to prevent H3 methylation. We found that all tested EZH2i doses affected lipid metabolism genes, ROS, and glutathione production. Transcript changes correlated with metabolic data, which suggested the effects of EZH2i on ferroptosis. We found that EZH2i dose-dependently increased SLC7A11/glutathione axis and glutathione peroxidase-4 (GPX4), required to counteract lipid peroxidation and ferroptosis. A GPX4 inhibitor synergized with EZH2i, making low doses - which otherwise do not affect cell viability - able to significantly reduce ACC cell growth in vitro and in vivo. Importantly, we found that the anti-ferroptosis defense mechanism induced by EZH2i is a common response for several aggressive tumor phenotypes, uncovering a general co-targetable mechanism that could limit EZH2i effectiveness. Correcting this antioxidant response by ferroptosis inducers may be a new combination therapy that will easily find clinical applications.

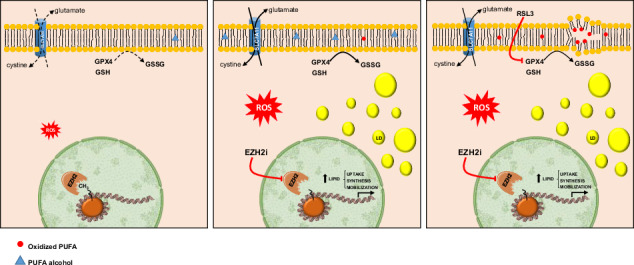

## Introductioncelldeat

Adrenocortical carcinoma (ACC) is a rare but very aggressive tumor, with 5-year survival rates ranging from 16 to 47%. The first-line therapy includes mitotane, a drug mainly used for its ability to control steroid production. Although mitotane and cytotoxic chemotherapy (etoposide, doxorubicin, and cisplatin) are often used in combination, the response rates are low, and discontinuation of mitotane-based therapy is commonly due to significant toxicity [1]. One of the effects elicited by mitotane is sterol *O*-acyltransferase 1 (SOAT1) inhibition, causing an excess of free cholesterol, responsible for endoplasmic reticulum stress and cell death [2]. After these findings, a selective and highly specific SOAT1 inhibitor, ATR-101 (Nevanimibe) was demonstrated effective in inducing adrenocorticotoxicity in vitro and in vivo [3]. Nevanimibe entered in clinical trial for the treatment of ACC, but the results were disappointing [4]. Other targeted therapies had been previously attempted, IGF1R monoclonal antibodies have been tested after the identification of IGF2 as a major driver of ACC progression, preclinical data were encouraging [5] but the clinical trial failed [6]. Despite several attempts, no effective targeted treatments have been, so far, identified.

Epigenetic regulators have gained considerable attention as their expression is deregulated in several cancers [7]. We have demonstrated that inhibition of the histone deacetylase SIRT1, potentiates mitotane action in inducing apoptosis in H295R human adrenocortical cancer cells [8]. Additionally, EZH2 (Enhancer Of Zeste 2 Polycomb Repressive Complex 2 Subunit), a methyl transferase controlling the epigenetic silencing of specific genes and/or microRNAs by trimethylating Lys27 on histone H3 [9], has been identified as the most deregulated histone modifier in adrenocortical cancer. EZH2 expression is associated with tumor proliferation and poor prognosis in ACC patients. EZH2 inhibition by the use of DZNep, a pharmacological inhibitor, or by RNA interference, was effective in reducing the growth and migration of H295R cells in vitro [10]. In addition to its activity as a histone modifier, it was shown that EZH2 works as a transcriptional inducer in ACC, cooperating with the transcription factor E2F1 to upregulate genes involved in cell cycle control and mitosis [11].

It must be pointed out that DZNep globally inhibits histone methylation and is not specific to EZH2 [12]. More specific inhibitors (here appointed as EZH2i) competing with the methyl donor S-adenosyl-methionine (SAM) [13] for the binding pocket of EZH2 have been developed. Among them, GSK126 can inhibit wild-type and mutant EZH2 with similar potency and shows high selectivity for EZH2 versus EZH1 or other methyltransferases [14]. However, results of a multicenter phase 1 clinical trial showed modest anticancer activity at tolerable doses in patients with advanced hematologic and solid tumors, and did not justify further clinical investigation [15]. Several other SAM-competitive inhibitors of EZH2 were developed including GSK343 [16], and tazemetostat (E7438/EPZ6438) [17]. GSK343 reduces H3K27me3 levels and inhibits EZH2 activity in breast and prostate cancer cells, but can only be used in vitro due to its high clearance [16]. Tazemetostat (TAZ) has improved potency and pharmacokinetics and was recently approved for the treatment of patients with metastatic or locally advanced epithelioid sarcoma who are not candidates for complete resection, and for patients with relapsed and refractory follicular lymphoma. New clinical trials are evaluating TAZ efficacy in patients with recurrent ovarian and endometrial cancer (NCT03348631) and solid tumors harboring mutations in the tumor suppressor gene ARID1A (NCT05023655).

EZH2i were shown to induce lipid accumulation in human adipocytes [18] and breast cancer cells [19]. More recently, it was demonstrated that GSK126 specifically increased the abundance of unsaturated fatty acids (FA) in several cancer cell models because of increased expression of stearoyl-CoA desaturase 1 (SCD1) and elongation of very-long-chain fatty acids-like 2 (ELOVL2). These genes are involved in the synthesis of monounsaturated and polyunsaturated FA (MUFA and PUFA), respectively. SCD1 knockdown increased cellular sensitivity to GSK126, supporting the idea that the upregulation of SCD1 by EZH2i may weaken its anticancer effect in solid tumors [20]. Despite the advance in deciphering mechanisms behind acquired resistance to EZH2 inhibitors [21], there are still several open questions.

MUFA and PUFA incorporated into phospholipids are highly susceptible to peroxidation in the presence of excessive amounts of reactive oxygen species (ROS). Cancer cells scavenge lipid hydroperoxides using glutathione (GSH) and glutathione peroxidase 4 (GPX4) [22]. Without an efficient peroxidation repair mechanism, cells undergo a death mechanism termed ferroptosis [23]. ACC are remarkably sensitive to ferroptosis and induction of this cell death mechanism has been proposed as a promising treatment approach for this type of cancer [24]. In this study, we investigated the dose-response effects of EZH2i on lipid metabolism, ROS production and antioxidant responses. We evidenced a remarkable change in cell metabolism and increased activity of the GSH/GPX4 axis, which represent vulnerabilities that can be targeted to potentiate the anticancer activity of EZH2i.

## Results

### ACC cells increase intracellular lipid content in response to EZH2 inhibitors

**Fig. 1:**
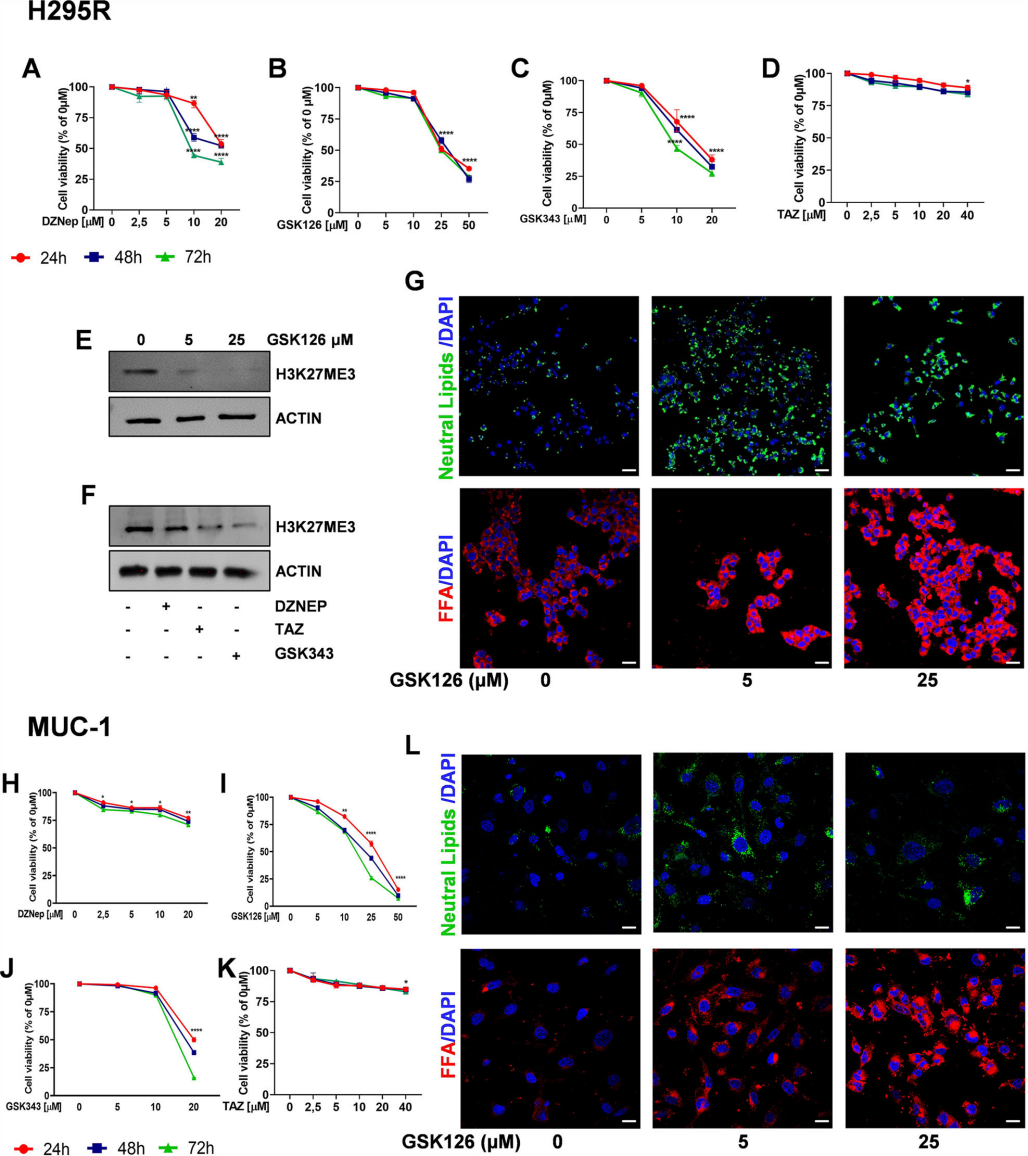
EZH2i affect cell viability and lipid content in ACC cells. ACC cells treated with DZNep, GSK126, GSK343, and Tazemetostat (TAZ). **A**–**D**, **H**–**K** Cell viability of H295R (**A**–**D**) and MUC-1 (**H**–**K**) was evaluated by MTT assay at 24, 48, and 72 h. (*n* = 3. Data were expressed as means ± SEM. *p < 0.05; **p < 0.01; ****p < 0.0001. **E**, **F** Total protein lysates from 48 h treated H295R cells were immunoblotted for H3K27me3. Actin was used as a loading control. **G**, **L** Confocal images of neutral lipids and free fatty acids (FFA) in 48 h treated H295R (**G**) and MUC-1 (**L**) cells. Nuclei were stained by DAPI (Scale bar 50 µm).

The general methyl transferase inhibitor DZNep, and three different EZH2 inhibitors, GSK126, GSK343, and tazemetostat (TAZ), collectively appointed as EZH2i, were used to treat two different ACC cell models: H295R and MUC-1. While H295R represents a mitotane-sensitive cell line, MUC-1 are a mitotane- and multidrug-resistant phenotype, with high migratory and metastatic potential [25]. Time course and dose-response evaluation on H295R cells, demonstrated that GSK126 and GSK343 were more effective in reducing cell viability than DZNep or TAZ (Fig. 1A–D). Importantly, the lower doses of GSKs did not produce significant inhibitory effects, despite being highly above the nanomolar concentrations required to inhibit the methyl transferase activity. In fact, the methylation status of histone 3 on lysine 27 (H3K27me3) was reduced by both low (5 µM) and high (25 µM) concentrations of GSK126 (Fig. 1E). Similarly, concentrations of DZNep, TAZ and GSK343, which did not reduce cell growth, decreased the methylation status of H3K27 (Fig. 1F). Lipid metabolism was shown to be involved in the limited EZH2i antitumor effects [19, 20]. H295R cells treated for 48 h with 5 µM and 25 µM of GSK126, were stained with specific dyes for neutral lipids (triglycerides and cholesterol esters), and free fatty acids (FFA) showed a remarkable increase in lipid accumulation (Fig. 1G), as in the presence of DZNep, GSK343, and TAZ (Fig. S1A). MUC-1 cells behaved very similarly to H295R cells in response to DZNep and EZH2i. The lower doses exerted minimal effects on cell viability (Fig. 1H–K), but caused a striking increase in lipid droplets (Figs. 1L and S1B), highlighting that EZH2i trigger a general but well addressed cell response in ACC, as seen in other tumor types [19, 20].

### EZH2 inhibition causes metabolic reprogramming in ACC cells

**Fig. 2:**
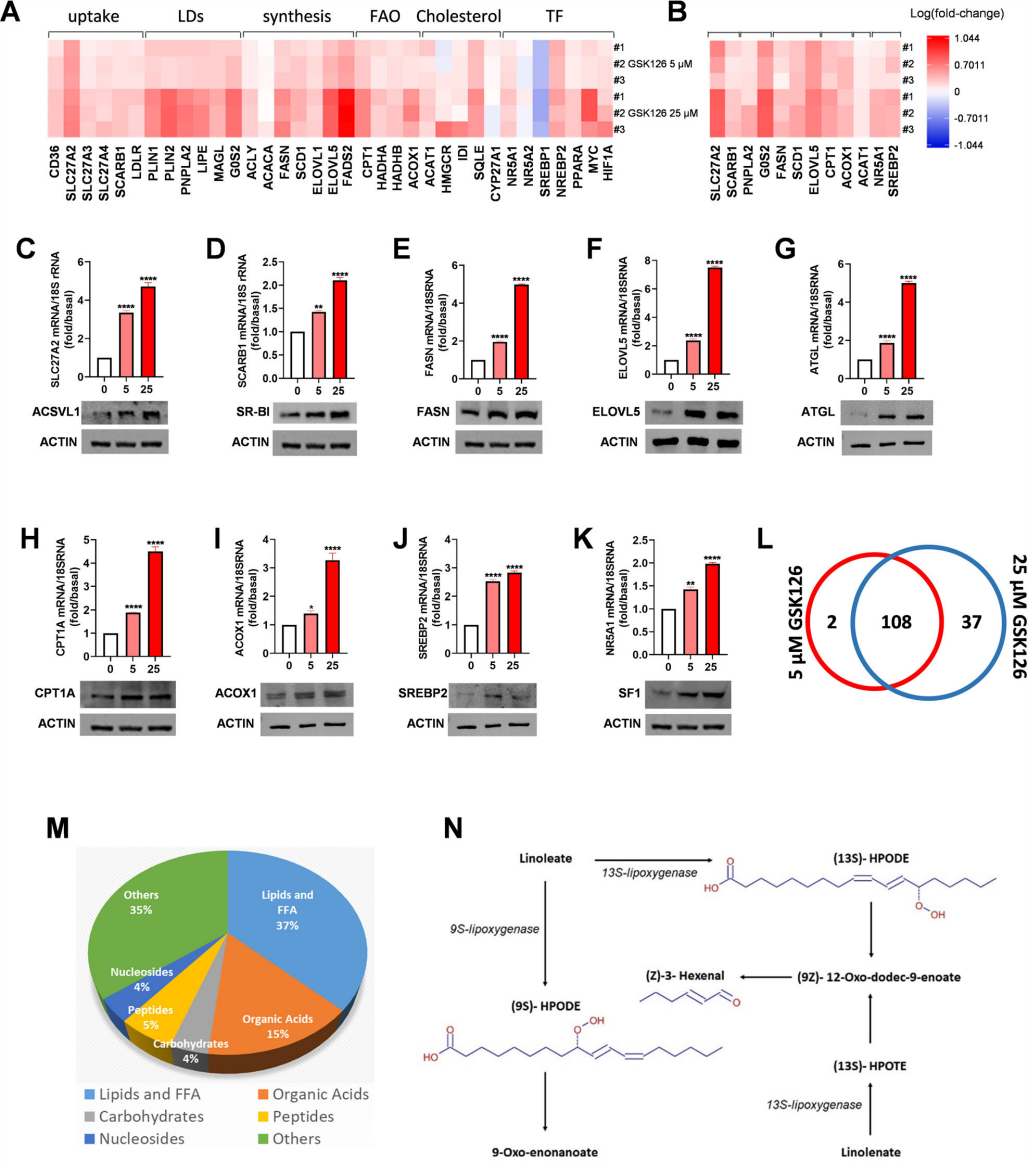
EZH2i change the metabolic profile of ACC cells. **A**, **B** Heatmap for mRNA expression of lipid metabolism genes in H295R cells after 48 h of GSK126 treatment. Data were the values from three separate RNA samples presented as log10 of fold change. LDs lipid droplets; FAO fatty acids oxidation, TF transcription factors. **C**–**K** QPCR graphs and western blots of representative genes from (**A**) (*n* = 3, means ± SEM *p < 0.05; **p < 0.01; ****p <0.0001). **L** Untargeted metabolomics analysis was performed on H295R cells treated with GSK126 (5 and 25 µM) for 48 h. Venn diagram represents statistically significant differentially abundant metabolites across 5 and 25 µM GSK126-treated cells. **M** Pie chart depicting the proportions of metabolite classes in the 108 common metabolites. **N** Schematic representation of linoleic acid oxidation pathways.

Using doses of GSK126 ineffective (5 μM) and effective (25 μM) in decreasing cell viability (Fig. 1B, I), we investigated the expression of lipid metabolism genes (Fig. 2A–C). A significant upregulation was found for genes involved in lipids uptake: CD36, SLC27A2, SLC27A3, SLC27A4, SCARB1, LDLR; FA mobilization from cellular stores: PLIN1, PLIN2, PNPLA2 (ATGL), LIPE (HSL), MAGL, and G0S2; FA synthesis: ACLY, ACACA, FASN, SCD1, ELOVL1, ELOVL5, and FADS2; FA oxidation (FAO): CPT1, HADHA, HADHB, and ACOX1. A similar change in the transcription of key genes from each metabolic group was also observed in MUC-1 cells treated with 5 and 25 µM GSK126 (Fig. 2B). We evaluated also the expression of genes related to cholesterol metabolism, and found that, with the exception of SQLE that was significantly upregulated by GSK126, HMGCR, IDI, and CYP27A1, were modestly affected. The increased expression of ACAT1, LDLR, and SR-B1 (from the SCARB1 gene) could account for the enhanced formation of cholesteryl esters (CE). The expression of transcription factors regulating the reported metabolic genes was also investigated. We observed an increase in the expression of SREBP2 (Fig. 2A, B), PPARα, Myc, and HIF1α (Fig. 2A). On the contrary, we found that GSK126 reduced SREBP1 mRNA levels (Fig. 2A). Of relevance, we observed an increase in NR5A1 mRNA, encoding for steroidogenic factor-1 (SF-1) (Fig. 2A, B), mainly involved in the expression of steroidogenic enzymes [26] and in cholesterol biosynthesis to support steroid synthesis [27]. However, SF-1 plays a role in energy metabolism by transcribing several key enzymes involved in glycolysis [28]. Glucose transporter gene SLC2A1, encoding for GLUT1, and the glycolytic genes HK1, HK2, PFKL, GAPDH, and LDHA were upregulated by both doses of GSK126 (Fig. S2A). Effects of DZNep and GSK343 on the expression of metabolic genes were also tested, evidencing the ability to change H295R cells transcriptome similarly to GSK126 (Fig. S2B). Furthermore, on MUC-1 cells DZNep and GSK343 produced effects similar to those observed on H295R (Fig. S2C). We confirmed that the upregulated mRNAs were also translated. Western blot analyses demonstrated that ACSVL1 (from SLC27A2 gene) (Fig. 2C), SR-BI (Fig. 2D), FASN (Fig. 2E), ELOVL5 (Fig. 2F), ATGL (Fig. 2G), CPT1A (Fig. 2H), ACOX1 (Fig. 2I), SREBP2 (Fig. 2J), and SF-1 (from NR5A1 gene) (Fig. 2K) protein content increased dose-dependently in the presence of GSK126. Altogether, these data indicate that doses of EZH2i, ineffective on cell viability, change cell transcriptome and proteome similarly to the concentrations which inhibit cell growth.

To link gene expression to metabolic changes, we performed an untargeted metabolomic analysis on H295R cells grown in the presence of 0 (control cells), 5 and 25 µM GSK126, through ultra-high performance liquid chromatography coupled to high-resolution mass spectrometry (UHPLC-HRMS). We were able to increase the number of identified molecules by tandem mass spectrometry and MS3 data through the Fragment Ion Search (FISh) scoring tool. We identified 110 and 145 metabolites significantly altered in 5 and 25 µM GSK126-treated cells, respectively, compared to control cells (adjusted *p* value <0.05 and Log2 Fold change >│1│) (dataset S1). A Venn diagram analysis identified 108 common metabolites that significantly changed in both 5 and 25 µM GSK126-treated cells (Fig. 2L), with the classes of those 108 metabolites shown in Fig. 2M. The metabolites extraction procedure allowed to identify many polar lipids and FFA (37% of total metabolites), even though the bulk of non-polar lipids was excluded from this analysis. The large amount of lipids and FFA whose amount was changed by GSK126 treatment, suggested a substantial reconfiguration of lipid metabolism.

Interestingly, in all GSK-treated cells we found decreased amount of 9-hydroperoxy-10,12-octadecadienoic acid ((9S)-HPODE), (2Z,4E)-2,18,18-trihydroxyoctadeca-2,4-dienoic acid and (7Z)-14-hydroxy-10,13-dioxoheptadec-7-enoic acid, which are products of hydroperoxidation of C18 and C17 unsaturated fatty acids. Linoleic acid (C18) is the most abundant PUFA in mammals and is a substrate for lipoxygenases (LOXs), which catalyze the formation of the corresponding hydroperoxides (9-HPODE and 13-HPODE) (Fig. 2N) [29–31], a hallmark of cell death by ferroptosis. The decrease in hydroperoxides in GSK126-treated cells could depend on either a decreased production of reactive oxygen species (ROS) or the induction of an antioxidant cell response. Of relevance, we observed that the two doses of GSK126 increased ROS content in H295R cells by 1.39- and 1.56-fold and in MUC-1 cells by 1.83- and 2.16-fold (Fig. S3). Then, GSK126 must trigger an antioxidant mechanism. The observation that GSK-treated cells have decreased levels of 3-carboxy-4-methyl-5-propyl-2-furanpropanoic acid (CMPF) (dataset S1), a ferroptosis-inducing factor [32], suggests that the antioxidant response could be engaged by tumor cells to sustain an anti-ferroptosis cell response.

### EZH2i-treated cells engage an antioxidant response to prevent ferroptosis

**Fig. 3:**
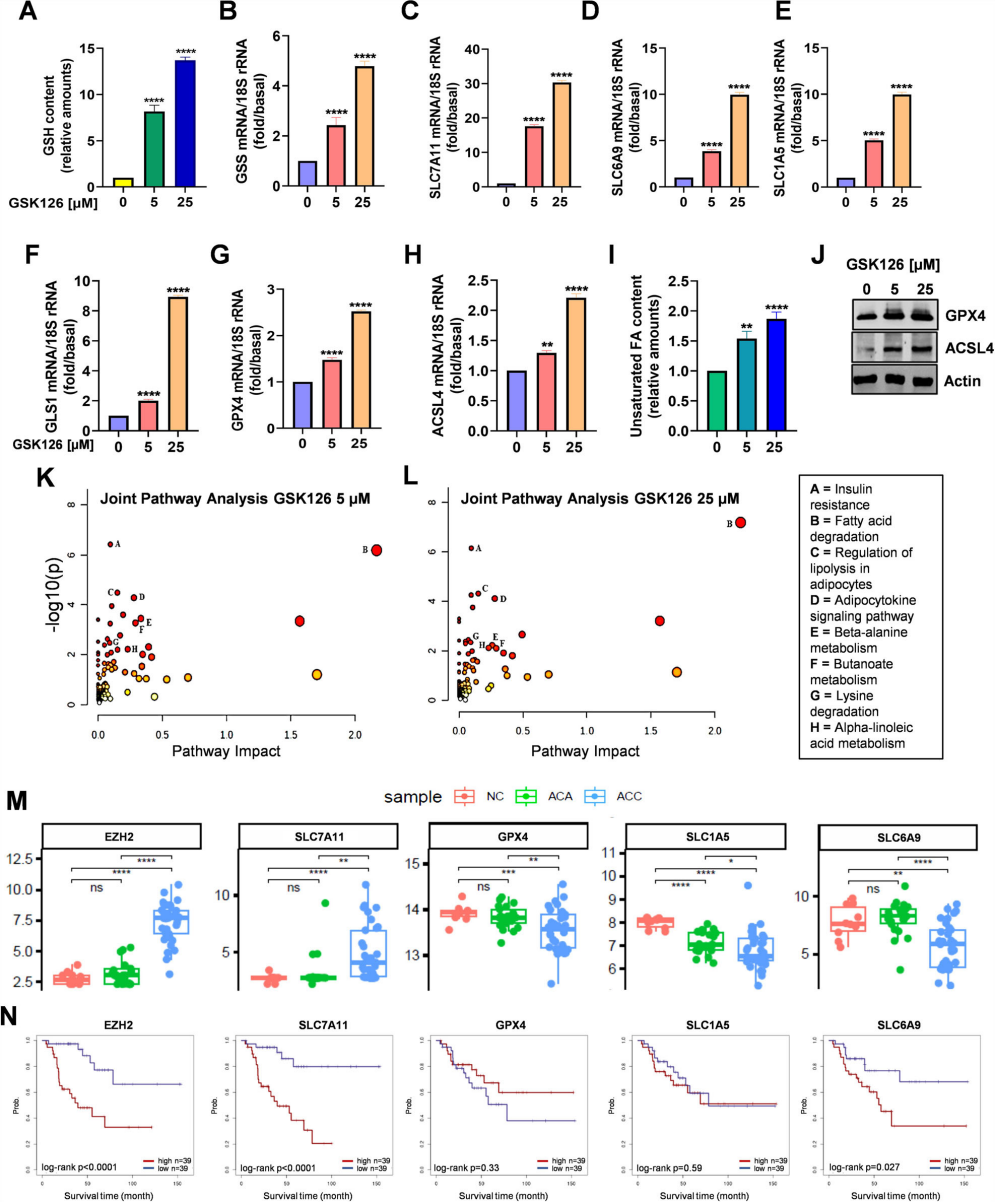
EZH2i activate antioxidant genes inversely correlated with ACC patients’ survival. H295R cells were treated for 48 h with GSK126. **A** Glutathione (GSH) content normalized to the number of cells. (*n* = 3, means ± SEM, ****p < 0.0001). **B***–***H** mRNA expression of genes related to ferroptosis. (*n* = 3, means ± SEM, **p < 0.01; ****p < 0.0001). **I** Unsaturated FA (MUFA and PUFA) quantification normalized to the number of cells. **J** Total protein lysates from 48 h treated H295R cells were immunoblotted for GPX4 and ACSL4. Actin was used as a loading control. **K**, **L** Overview of the pathway enrichment analysis based on both transcripts and metabolites with statistically significant differences in GSK126-treated H295R cells. Only pathways altered in both treatments and with –log10(p) >2 are shown. **M** Differential expression analysis of selected genes among normal adrenal (NC), adrenocortical adenomas (ACA), and adrenocortical carcinomas (ACC) from microarray cohort [61]. **N** Overall survival depicted by Kaplan–Meier plots for each of the selected genes in the ACC-TCGA dataset [63]. “Low” and “High” level expression groups are indicated by blue and red colors, respectively. Log-rank *p* values are shown at the bottom of each plot.

As the most abundant antioxidant in the cell [33], glutathione (GSH) content was measured in H295R cells. A robust dose-dependent increase, ranging from 9- to 14.4-fold over basal levels was evidenced (Fig. 3A). GSH is synthesized from the amino acids glutamate, cysteine, and glycine. We evidenced an upregulation in the glutathione synthase gene (GSS) (Fig. 3B) which catalyzes the addition of glycine to the glutamate-cysteine dipeptide. The three amino acid transporters SLC7A11 (cystine) (Fig. 3C), SLC6A9 (glycine) (Fig. 3D), and SLC1A5 (glutamine) (Fig. 3E) were also upregulated by GSK126. Particularly significant was the effect on SLC7A11, upregulated 17.7-fold by 5 μM and 31.6-fold by 25 µM GSK126 (Fig. 3C). Before being used for GSH synthesis, glutamine needs to be converted into glutamate through the action of glutaminase (GLS1), that we also found dose-dependently upregulated in response to GSK126 (Fig. 3F) and to GSK343 but not to DZNep (Fig. S2B), evidencing specific differences between selective EZH2 inhibitors and drugs acting as general methyltransferases inhibitors. GSH can be used by peroxidases (GPXs) to repair peroxidized lipids. We found that GSK126 increased mRNA and protein levels of GPX4 (Fig. 3G, J), which is essential for repairing peroxidized lipids and preventing ferroptosis. Lipid peroxidation is a free radical oxidation of lipids containing carbon-carbon double bond(s) such as MUFA and PUFA, causing cell thinning and, ultimately, rupture. PUFA, that we found elevated in response to GSK126 (Fig. 3I), are incorporated into phospholipids by ACSL4, that we also found upregulated (Fig. 3H, J). We then performed an integrated analysis using the differentially expressed metabolic gene transcript data and the metabolomics data from 5 and 25 μM GSK126-treated H295R cells through joint pathway enrichment analysis (Fig. 3K, L). The main altered pathways under both treatments were related to lipid metabolism. In 25 µM GSK126-treated cells, the alteration of glutathione metabolism and ferroptosis pathway was confirmed. The complete lists of significant pathways in joint pathway analysis are in Tables S1, S2.

GSK126 induced similar responses in MUC-1 cells (Fig. S2D), highlighting ACC cell ability to counteract EZH2i-induced toxic effects by efficiently embracing the SLC7A11/GSH/GPX4 axis. This pathway is recognized as the primary defense mechanism against ferroptosis. Accordingly, gene expression analysis indicated that EZH2 and SLC7A11 are upregulated in ACC in comparison to normal adrenal (NC) and adrenocortical adenoma (ACA). On the contrary, expression of SLC1A5, SLC6A9 and GPX4 was downregulated in ACC (Fig. 3M) and could explain the sensitivity to ferroptosis inducers [24]. Additionally, high expression levels of EZH2, SLC7A11, and SLC6A9 are associated with decreased overall survival in ACC, while GPX4 does not have an impact (Fig. 3N).

To interrogate whether ferroptosis-related genes would provide additional information to EZH2 in predicting overall survival in ACC-TCGA patients, we used bivariate Cox regression models, including the expression of EZH2 and candidate genes in each model. Results are summarized in Table S3. To assess whether the addition of a second variable (ferroptosis genes) improved the fitness of the univariate model (EZH2), we performed likelihood-ratio tests comparing the univariate model (EZH2 only) and each of the bivariate models. For some of the models, the addition of a second variable (ferroptosis gene), improved the fitness of the univariate model, suggesting that it provided further information relevant for overall survival. To visualize this effect, Kaplan–Meier curves were obtained, dividing samples into four groups, based on the median expression of EZH2 and each of the ferroptosis genes (Fig. S4A–F). Log-rank tests of each pair-wise comparison were performed. In fact, as demonstrated in the Kaplan–Meier curves, the addition of further stratification according to the expression of ferroptosis-related genes, can further improve risk stratification by identifying a subgroup of EZH2-high patients with very high risk.

### EZH2 inhibition sensitizes tumor cells to ferroptosis inducers

**Fig. 4:**
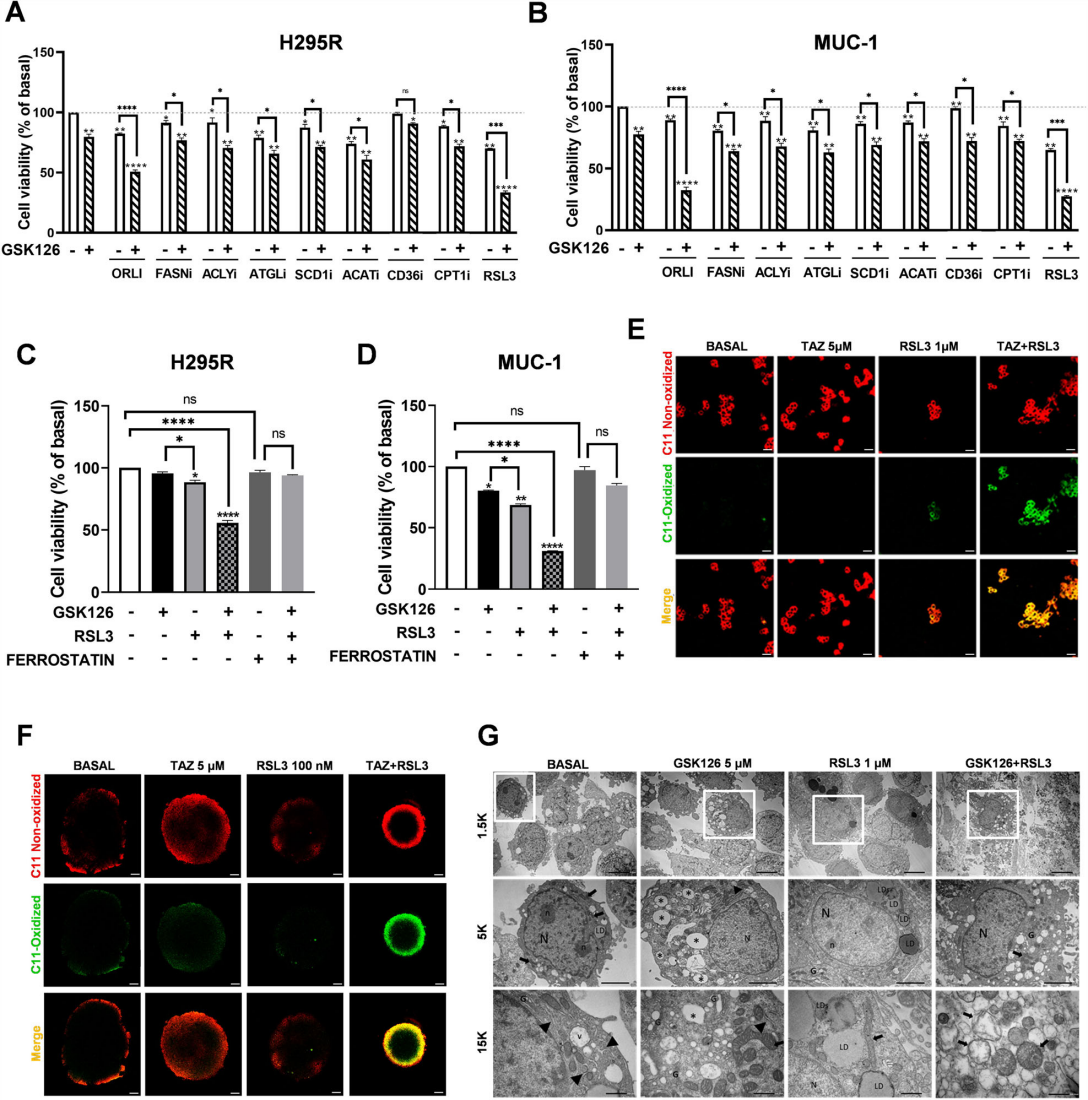
Low doses of EZH2i synergize with ferroptosis inducers. **A**, **B** ACC cells treated for 48 h with GSK126 (5 μM) alone or in combination with inhibitors for lipid metabolism enzymes: Orlistat (ORLI 100 μM), FASNi (G28UCM, 1 μM), ACLYi (SB204990, 50 μM), ATGLi (ATGLstatin, 50 μM), SCD1i (A959572, 5 nM), ACATi (Avasimibe, 2.5 μM), CD36i (Sulfosuccinimidyl oleate, 10 μM), and CPT1i (Etomoxir, 10 μM). **C**, **D** ACC cells treated for 48 h with GSK126 (5 μM) in the presence or absence of RSL3 (1 µM, for the last 4 h) and Ferrostatin (1 µM). (*n* = 3. Data were expressed as means ± SEM. *p < 0.05; **p < 0.01; ***p < 0.001; ****p < 0.0001. ns not significant). **E**, **F** Confocal images of lipid peroxidation detected by BODIPY-C11 fluorescent dye in 2D (**E**) and 3D H295R cultures (**F**) (scale bar 50 µm). **G** Electron micrographs. Nucleus (N), nucleolus (n), mitochondria (black arrows), endoplasmic reticulum (black arrowheads), Golgi apparatus (G), electron transparent vesicles and vacuoles (v), lipid-like vacuoles of low electron density (*), lipid droplets (LD) (scale bars: 5 µm, 2 µm and 500 nm).

We next investigated if the lipid metabolism gene signature selected by EZH2i could prevent their effectiveness against tumor cell growth. Then, co-targeting the identified upregulated genes (Figs. 2 and S2) would make the low doses of EZH2i effective in reducing ACC cell growth. We performed MTT assay on H295R (Fig. 4A) and MUC-1 (Fig. 4B) combining GSK126 5 µM with inhibitors for FASN, ACLY, ATGL, SCD1, ACAT1, CD36 and CPT1. For the tested inhibitors, we used doses in the known IC50 ranges, evidencing minimal effects on cell viability. A more pronounced inhibition was observed following combined treatment, however never reaching synergistic effects with GSK126. Indeed, a synergistic inhibitory effect was evidenced only for the combination GSK126 + orlistat, that caused more than 50% inhibition on cell viability in both cell lines (Fig. 4A, B). We also evaluated GPX4 as a possible co-target, evidencing a significant synergistic effect of GSK126 combined with RSL3, a GPX4 inhibitor and ferroptosis inducer (Fig. 4A, B). Accordingly, the use of ferrostatin, a ferroptosis inhibitor, reversed GSK126 + RSL3 effects on cell viability (Fig. 4C, D). To further prove ferroptosis as a cell death mechanism, we evaluated the amount of lipid peroxides in cellular membranes using a BODIPY-C11 probe. A clear shift from red to green signal characterized ACC cells under GSK126 and RSL3 combined treatment (Figs. 4E and S5), indicating the loss of the membrane repair capacity. In H295R cells grown as spheroids, the combined treatment was effective in reducing sphere size and in inducing a remarkable lipid peroxidation in the proliferating layer of the 3D structure (Fig. 4F). Ultrastructural assessment revealed that control cells exhibited intact membranes and cytoplasm containing mitochondria, a well-developed Golgi system, abundant rough endoplasmic reticulum (ER) and lipid droplets (LDs) that appeared as moderately large bodies with a spherical shape and a homogenous internal structure of low electron density. Electron-transparent vesicles and vacuoles of various sizes can be seen throughout the cytoplasm. The nuclei were uniform with finely dispersed chromatin and clearly visible nucleoli (Fig. 4G). In GSK126-treated cells, the cytoplasm became more electron transparent, and vacuoles of low electron density greatly increased in number. The ER showed a regular arrangement and the nucleus was morphologically well preserved with an intact nuclear envelope. Mitochondria tended to elongate and the number of Golgi bodies increased, becoming much enlarged to form vesicles, which in sections appeared as electron-transparent spheres (Fig. 4G). RSL3-treated cells showed a regular arrangement with a normal nuclear shape and a continuous plasma membrane. LDs increased in number with variable electron density, ranging from electron lucent to dark gray. In the longitudinal section, mitochondria maintained their tubular shape and frequently appeared to be longer compared to those of control cells (Fig. 4G). The vast majority of GSK126 + RSL3-treated cells exhibited severe damage, evidenced by extensive swelling and disorganization of cytoplasmic organelles and loss of plasma membrane integrity. Clear spaces or vacuoles appeared in the cytosol that became electron lucent and the nuclear chromatin was rarefied. Mitochondria were swollen and contained disorientated and disintegrating cristae located at the periphery of the organelle. The mitochondrial matrix was less dense than in control cells (Fig. 4G). All these ultrastructural changes are features strictly related to ferroptosis, and can be observed only in the combined treatment, evidencing that EZH2i-treated cells become more sensitive to ferroptosis inducers.

### A combination of EZH2i with ferroptosis inducers is an effective anticancer therapeutic approach

**Fig. 5:**
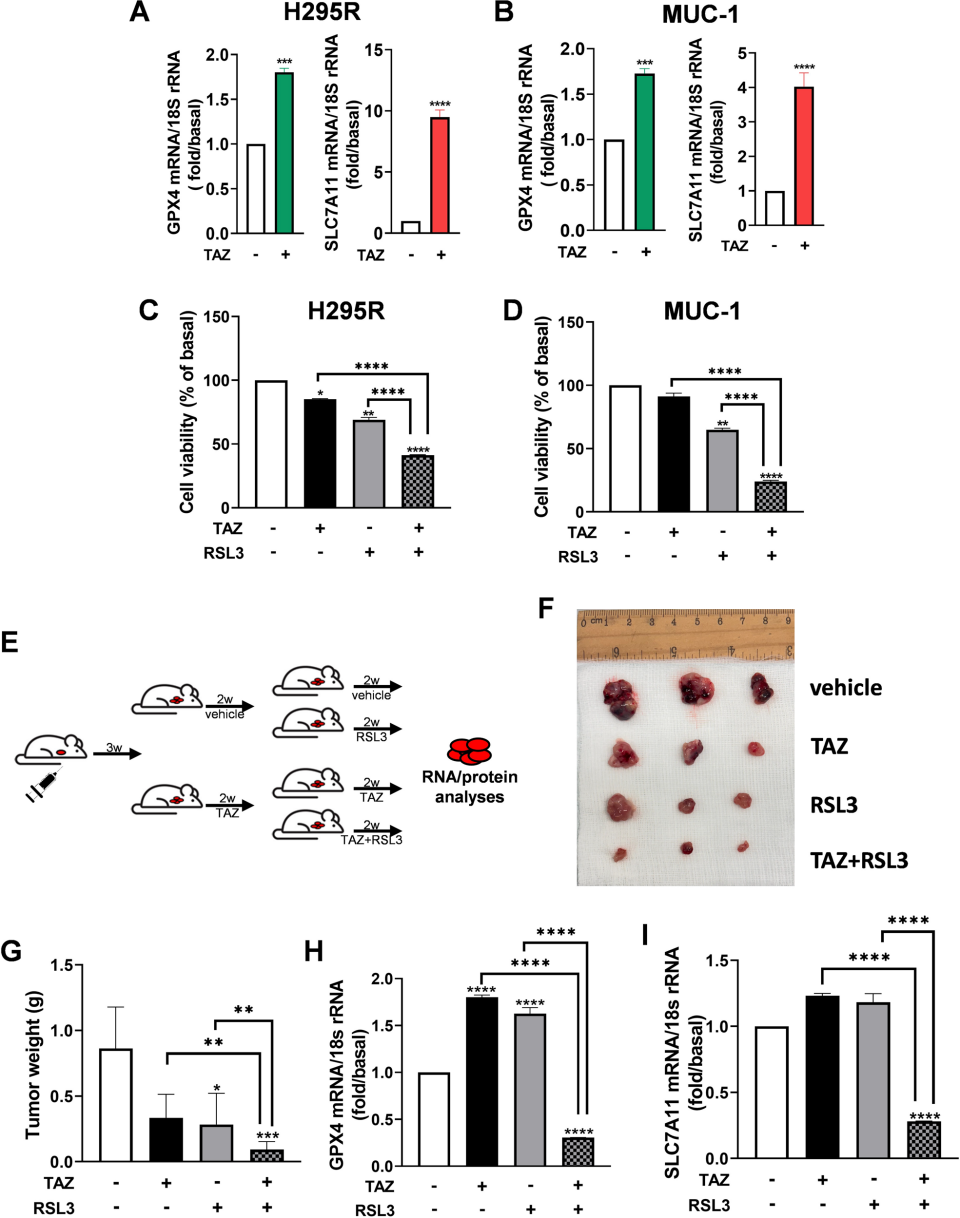
In vitro and in vivo synergistic effects of tazemetostat and ferroptosis inducers. **A**, **B** mRNA expression of GPX4 and SLC7A11 in ACC cell lines treated for 48 h with tazemetostat (TAZ 5 µM). **C**, **D** Cell viability of tumor cell lines treated for 48 h with TAZ (5 μM) and RSL3 (1 µM, for the last 4 h) and their combinations. **E** Schematic representation of xenograft experiment setup. **F**, **G** Images of explanted xenograft tumors (**F**) and relative tumor weight graphs (**G**). **H**, **I** mRNA expression of GPX4 and SLC7A11 in xenografts tumor samples. Data were expressed as means ± SEM. *p < 0.05; **p < 0.01; ***p < 0.001 ****p < 0.0001.

Having discovered that the GSH/GPX4 antioxidant response limits EZH2i antitumor effects in ACC, gives the possibility to turn a limitation into a therapeutic advantage, using EZH2i with ferroptosis inducers as a combination strategy. We tested the effects of tazemetostat (TAZ), an EZH2i used in the clinic, on GPX4 and SLC7A11 gene expression in H295R and MUC-1 cells, evidencing a significant increase in the expression of both genes (Fig. 5A, B). The enhanced antioxidant response sensitized tumor cells to RSL3, resulting in a synergistic inhibitory effect on cell viability (Fig. 5C, D).

To further prove the efficacy of TAZ + RSL3 treatment, we tested their effects on H295R xenografts. Tumors were left to grow, and three weeks later, animals were divided into two groups to receive either vehicle (methyl-β-ciclodextrin) or TAZ (10 mg/kg). Two weeks later, each of the two groups was split into two subgroups: one continued to receive the initial treatment and the second in addition received RSL3 (3 mg/kg) (Fig. 5E). The combination of the two inhibitors had a dramatic effect on tumor growth (Fig. 5F), showing a 90% reduction in tumor weight (Fig. 5G). TAZ increased GPX4 (Fig. 5H) and SLC7A11 (Fig. 5I) mRNA in vivo as seen in vitro (Fig. 5A).

**Fig. 6:**
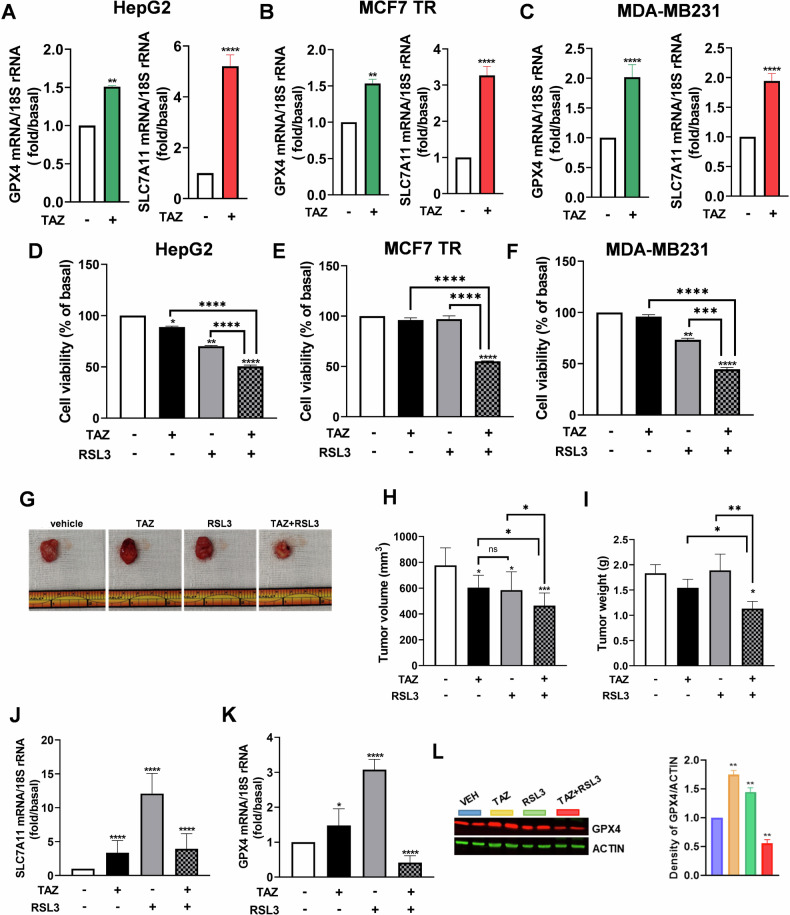
Tazemetostat combined with ferroptosis inducers is an effective treatment for different tumor types. **A**–**C** mRNA expression of GPX4 and SLC7A11 in cell lines from different tumor types treated for 48 h with tazemetostat (TAZ 5 µM). **D**–**F** Cell viability of tumor cell lines treated for 48 h with TAZ (5 μM) and RSL3 (1 µM, for the last 4 h) and their combinations. **G**–**I** Representative images of explanted MDA-MB231 xenografts (**G**) and relative graphs of tumor volume (**H**) and weight (**I**). **J**, **K** mRNA expression of GPX4 and SLC7A11 in xenografts tumor samples. **L** Protein expression and relative quantification of GPX4 in tumor samples. Data were expressed as means ± SEM. *p < 0.05; **p < 0.01, ***p < 0.001;  ****p < 0.0001.

Having discovered that the GSH/GPX4 antioxidant response limits EZH2i efficacy in ACC, we hypothesized that a similar mechanism dampened their effects in other tumor types. We used cell models for hepatocellular carcinoma (HepG2), tamoxifen-resistant estrogen receptor-positive breast cancer (MCF7/TR), triple-negative breast cancer (MDA-MB231) and tested the effects of tazemetostat (TAZ) on GPX4 and SLC7A11 gene expression evidencing a significant increase of both genes in all tumor cell models (Fig. 6A–C). As for ACC cells, we observed a synergistic inhibitory effect on cell viability (Fig. 6D–F).

To further prove the efficacy of TAZ + RSL3 treatment, we used MDA-MB231 cells for in vivo studies. These cells are an aggressive model of TNBC, a breast cancer subtype with adverse clinical outcomes and no available targeted therapy. As for ACC [10], expression of EZH2 in TNBC has been shown to correlate with metastatic phenotype and poor prognosis [34]. Preliminary in vitro data confirmed that, as for ACC models, MDA-MB231 cells increase the neutral lipids and free fatty acids content in response to EZH2i (Fig. S6A). Cell viability was reduced by GSK126 only at concentrations far above the IC50 value (Fig. S6B), while TAZ had minimal effects (Fig. S6C) but dose-dependently increased GPX4 expression (Fig. S6D). Treatment with increasing doses of RSL3 decreased cell viability, evidencing the sensitivity of these cells to the ferroptosis inducer (Fig. S6E).

Untargeted metabolomics analysis was performed on TNBC (MDA-MB231) cells treated with 5 and 25 µM GSK126 to give an insight into metabolic rewiring. We found 60 metabolites whose amounts were significantly changed in 5 µM GSK126-treated cells and 175 metabolites significantly altered in 25 µM GSK126-treated cells compared to control cells (adjusted *p* value <0.05 and Log2 fold change >│1│) (dataset S2) 55 metabolites were altered by both treatments (Fig. S7A). The altered polar lipids and FFA were 35% of total metabolites in 5 µM GSK126-treated cells and 30% in 25 µM GSK126-treated cells, similar to what we found in H295R cells. In addition, most of the compounds differentially expressed in GSK126-treated MDA-MB231 cells were different from the compounds whose amount was changed in GSK126-treated H295R cells, only a few altered metabolites being in common between the two cell lines. However, the joint pathway enrichment analysis highlighted the alteration of lipid metabolism (alpha-linolenic acid metabolism, adipocytokine signaling pathway, and fatty acid degradation), glutathione metabolism, and insulin resistance as in 25 µM GSK-treated H295R cells (Fig. S7B and Table S4). These data confirm that key features evidenced for ACC cells in response to EZH2i are also reproduced in triple-negative BC cells, supporting the use of this model to test the efficacy of TAZ + RSL3 in in vivo studies following the same treatment set-up as for H295R xenografts (Fig. 5E). Tumor explant showed a reduction in their size in the drugs-treated groups, which was more evident for tumors from mice treated with TAZ + RSL3 (Fig. 6G, H). These observations agree with a reduced tumor weight (Fig. 6I). In addition, TAZ increased SLC7A11 (Fig. 6J) and GPX4 (Fig. 6K, L) in vivo as seen in vitro (Fig. 6C).

## Discussion

EZH2, a histone methyl transferase subunit of a Polycomb repressor complex, is recurrently mutated or highly expressed in several cancers, including ACC [10] and its deregulation is associated with tumor proliferation and worse patient outcomes [35]. A series of EZH2 inhibitors have been discovered and tested in clinical trials, among them GSK126, tazemetostat, CPI-1205, PF-06821497, and SHR2554 [13]. Clinical trials on solid tumors have not lived up to the high expectations, and have failed to demonstrate any substantial benefit at the tolerable doses. However, in 2020 tazemetostat was approved by the US Food and Drug Administration (FDA) for the treatment of epithelioid sarcomas and follicular lymphomas.

We tested four different EZH2i (DZNep, GSK126, GSK343, and TAZ) for their effects on the cell viability of mitotane-sensitive, H295R, and mitotane-resistant, MUC-1 cells [25]. EZH2i exerted a pronounced cell growth inhibition at doses much higher than those requested to inhibit EZH2 activity, as seen for other tumors [36]. Surprisingly, TAZ was ineffective at all tested doses. A recent study shed some light on the limited efficacy of EZH2i pointing to an increased lipid metabolism as an important event impairing their antitumor effects. Zhang et al. [20]. reported an increase in lipid content in melanoma, hepatocellular carcinoma, and colorectal cancer cells at doses that modestly affected cell proliferation, but profoundly changed cell transcriptome and metabolome. Genes involved in lipid metabolism were significantly upregulated, and their expression correlated with an accumulation of a fatty acid pool closely associated with tumor progression [20]. Similarly, our ACC cell models showed a dramatic increase in both neutral lipids and FFA content in response to EZH2i.

We evaluated all the mechanisms used by cells to increase their lipid content. EZH2i-treated cells enhanced FA synthesis, as evidenced by an increased expression of FASN, SCD1, FADS2, and ELOVL5, providing saturated FA, MUFA, and PUFA. Quantification of unsaturated FA proved the association between rearranged gene expression and cell response. Additionally, genes for cholesterol and FA uptake, such as SR-BI and SLC27A2, were upregulated. EZH2i increased genes related to FA mobilization from cellular stores such as ATGL, responsible for triacylglycerol (TAG) catabolism whose activity could be prevented by the concomitant upregulation of its inhibitor G0S2, potentially explaining the increased LDs content. Genes related to mitochondrial and peroxisomal FA β-oxidation (FAO), CPT1, and ACOX1, were also upregulated by EZH2i. The increased peroxisomal activity was further supported by the increased expression of SLC27A2, which also localizes on peroxisomal membranes. In C. elegans, dietary MUFAs upregulate the number of lipid droplets and peroxisome, creating an organelle network with a central role in lipid homeostasis, associated with improved lifespan [37]. This adaptive metabolic response could occur in EZH2i-treated cells where a similar organelle network might promote tumor cell survival.

The change in lipid transcriptome prompted us to analyze the expression of key transcription factors [38]. We found that EZH2i upregulated c-Myc, regulating ACLY and FASN gene transcription [39]. Additionally, we observed an increased expression of SREBP2, which can also mediate transcriptional activation of many of the identified lipid metabolism genes. By contrast, SREBP1 mRNA was downregulated as a consequence of the increased levels of MUFA/PUFA, which are known inhibitors for SREBP1 [40]. PUFA are instead activators for PPARα, the major regulator of fatty acid oxidation, that we also found upregulated by EZH2i, and could account for the upregulated FAO genes. In addition to sterol-sensing regulation, SREBP2 can undergo ROS-dependent activation [41]. The EZH2i-dependent ROS increase is ultimately responsible for HIF1α activation, a known regulator of glycolytic genes. Extremely relevant is our observation that GSK126, at doses ineffective (5 μM) and effective (25 μM) in reducing cell viability, upregulated SF-1, a transcription factor with a central role in adrenal development and steroidogenesis [42], also involved in the transcriptional regulation of cholesterol synthesis [27] and glycolysis-related genes [28]. In ACC, SF-1 is involved in the regulation of transcripts involved in the cell cycle control, apoptosis, and cell adhesion to the extracellular matrix [43]. Our discovery of SF-1 upregulation uncovers an unwanted side effect that could limit the benefits of using EZH2i as monotherapy for the treatment of ACC.

Metabolomics data revealed a consistent activation of glutathione metabolism, suggesting the activation of a protective mechanism for the maintenance of cellular redox homeostasis, which requires different factors, including GSH. GSH amounts were increased by GSK126 as a consequence of upregulation in the expression of glutathione synthetase (GSS), and amino acid transporters SLC1A5, SLC6A9 and SLC7A11, providing substrates for GSH synthesis. GSH is then used by GPX4 to repair peroxidized lipids which, indeed, were reduced in EZH2i-treated H295R cells. The antioxidant SLC7A11/GPX4 axis repairs the peroxidation of PUFA-containing phospholipids [44], counteracting ferroptosis, a type of programmed cell death [45]. Indeed, tumor cells may significantly enhance their oxidative stress defense ability by upregulating the SLC7A11/GSH/GPX4 axis, achieving drug resistance and survival to ferroptosis [46]. Then, activation of this mechanism in response to EZH2i may hinder their effectiveness in cancer therapy. However, having decrypted the metabolic reprogramming gave us the possibility to test new combination therapies, turning a protective mechanism into a vulnerability. RSL3, a GPX4 inhibitor (GPX4i), at a dose that did not have much effect when used alone, produced a synergistic effect with GSK126 in reducing cell growth. The combination with RSL3 was more effective than any co-treatment with several inhibitors for the regulated genes, including FASN, ACLY, ATGL, SCD1, ACAT1, CD36 and CPT1. The most pronounced inhibition was reached using FASN inhibitor, orlistat, that however is a drug with off-target effects upon eight proteins with a proven role in tumor biology [47]. Three of the identified proteins, RPL7a, RPL14, and RPS9, are ribosomal proteins involved in protein synthesis, control of cellular transformation, tumor growth, aggressiveness, and metastasis. This might explain the more pronounced effects of orlistat over G28UCM, a more specific FASN inhibitor. SCD1 inhibitors are currently tested in the preclinical setting to induce ferroptosis [48, 49], considering that SCD1-derived MUFA, are incorporated in cell membranes providing a robust protection from ferroptosis [49, 50]. Then, Zhang et al [20], by co-targeting EZH2 and SCD1, likely limited cell ability to protect themselves from ferroptosis. In our co-treatment experiments, the dose of SCD1i was chosen in the nanomolar range to limit its inhibitory effects as a single agent, but it cannot be excluded that higher concentrations could produce a more significant inhibition on cell growth. Xenograft experiments using H295R cells evidenced a potent antitumor effect of TAZ in combination with RSL3. Importantly, ACC cells are very sensitive to ferroptosis inducers, and targeting this mechanism has been indicated as a valid therapeutic alternative to mitotane [24], which exhibits high toxicity and still limited therapeutic responses. We evidenced that the anti-ferroptotic mechanism might be considered a common response, since it is initiated by different cancer cell types exposed to EZH2i. The combination of Tazemetostat with RSL3, potentiated the anticancer activity of the single agents on cell models for hepatocellular carcinoma, tamoxifen-resistant, and triple-negative breast cancer. The latter cell model, as for ACC, still lacks a targeted therapy.

Our metabolomic data on TNBC cells exposed to GSK126 evidenced a pronounced enrichment in their MUFA/PUFA pool, targets of the GSH/GPX4 repair system against lipid peroxidation. Having demonstrated that EZH2i ignites a common defense mechanism in cancer cells, other than ACC models, widens the translational application of our study. The effectiveness of the combined treatment was further demonstrated by xenograft studies, evidencing a significant decrease in tumor weight and volume associated with an enhanced expression of GPX4 and SLC7A11 in response to TAZ. TCGA database analysis further highlighted the relevance of the antioxidant system in ACC. The expression of SLC7A11 negatively correlates with ACC patients’ survival, as seen for several cancers, such as invasive breast cancers [51] and hepatocellular carcinoma, among others [52]. The Kaplan–Meier curves and the Cox regression models show that SLC7A11 adds further stratification to EZH2 subgroups in terms of overall survival. In particular, EZH2-high/SLC7A11-low have better outcomes than EZH2-high/SLC7A11-high. This observation further supports the clinical relevance of targeting this antioxidant system.

In conclusion, this work indicates that ACC cells respond to EZH2i by increasing FA and ROS content. This occurs at both low doses, slightly affecting cell growth, and at high doses, causing a significant decrease in cell viability. These events are concomitant with the activation of a robust cellular antioxidant response, characterized by increased expression of enzymes and amino acids membrane transporters needed for glutathione synthesis. GSH is used as a cofactor by GPX4, which reduces the peroxidized phospholipids and prevents ferroptosis. However, while in the presence of lower concentrations of EZH2i, the amount of ROS can be kept under control and handled by tumor cells to avoid lipid peroxidation, the use of higher EZH2i concentrations increases FA and ROS content to the point of becoming toxic to cells, impairing their viability. Translating these in vitro observations into a potential patient’s response, the concentrations of EZH2i needed to produce an effect would be too high and too toxic. Our data suggest that the route to be followed to lower EZH2i doses is a combined therapy with ferroptosis inducers. Of note, in the combination settings, ferroptosis inducer dose can also be decreased, and toxicity avoided. This approach can be a valid means to decrease EZH2i concentration and improve their therapeutic efficacy for several tumor types.

## Materials and methods

### Cell cultures

All of the cell lines used were grown in a humidified 5% CO_2_ at 37 °C and obtained from the American Type Culture Collection (ATCC, Rockville, MD). MUC-1 cells were obtained from Constanze Hantel (University of Zurich-Switzerland) [53]. H295R and MUC-1 cells were cultured as previously described [54]. Tamoxifen-resistant MCF7 (MCF7-TR) [55] cells were cultured in DMEM/F-12 (GIBCO 31330-038) + 2 mM l-glutamine + 1% penicillin/streptomycin (P/S) + 5% fetal bovine serum (FBS) + Tamoxifen 1 µM; Basal-like breast cancer MDA-MB-231 cells were cultured in DMEM/F-12 (GIBCO 31330-038) + 2mM l-glutamine + 1% P/S + 10% FBS. Hepatocellular carcinoma HepG2 cells were cultured in DMEM (high glucose, SIGMA-Aldrich D5671) + 2 mM l-glutamine + 1% P/S + 10% FBS. For experiments on 3D cultures, a single-cell suspension of H295R cells was prepared using 1X trypsin-EDTA solution (SIGMA) followed by manual disaggregation (21-gauge needle). Cells were seeded in non-adherent conditions for 4 days, and treated for 4 additional days.

### Western blot analysis

Total cell lysates were prepared in RIPA buffer (50 mM Tris-HCl, 150 mM NaCl, 1% NP-40, 0.5% sodium deoxycholate, 2 mM sodium fluoride, 2 mM EDTA, 0.1% SDS and a mixture of protease inhibitors). Protein concentration was determined by the Bradford (BIO-RAD) method and equal quantities were subjected to Western blot analysis. SDS-PAGE-separated proteins were electroblotted onto a nitrocellulose membrane. Blots were incubated overnight at 4 °C with the following primary antibodies:

anti-H3K27me3 (A15024; 1:1000; Invitrogen); anti-ACSL4 (MA531543; 1:1000; Invitrogen); anti-ACSVL1 (sc-393906; 1:1000; Santa Cruz Biotechnology); anti-ATGL (sc-365278; 1:1000; Santa Cruz Biotechnology); anti-CPT1A (sc-393070; 1:1000; Santa Cruz Biotechnology); anti-ELOVL5 (PA5115176; 1:1000; Invitrogen); anti-FASN (sc-48357; 1:1000; Santa Cruz Biotechnology); anti-GPX4 (MA532827; 1:1000; Invitrogen); anti-SR-B1 (ab52629; 1:1000; Abcam); anti-ACOX1 (sc-517306; 1:1000; Santa Cruz Biotechnology);anti-SREBP2 (sc-271615; 1:1000; Santa Cruz Biotechnology); anti-SF-1 (07-618 Merck Millipore). Anti-beta actin (ab8226; 1:1000; Abcam) was used as a loading control. All antibodies were incubated with appropriate horseradish peroxidase-conjugated secondary antibodies for 1 h at room temperature. Immunoreactive bands were detected by the ECL Western blotting detection system (Santa Cruz Biotechnology, sc-2048). Immunoreactive bands were acquired by the iBright detection system (Thermo Fisher).

### Cell viability assay

Cell viability was measured using the 3-(4,5-dimethylthiazol-2-yl)-2,5-diphenyltetrazolium bromide (MTT) (Sigma-Aldrich) colorimetric assay. Cells were plated in 48-well plates and treated with DZNEP, GSK126, GSK343, tazemetostat, orlistat, G28UCM, SB204990, ATGLstatin, A959572, Avasimibe, sulfosuccinimidyl oleate (SSO) etomoxir, and ferrostatin (all from MedChemExpress). RSL3, a GPX4 inhibitor, was added for 4 h following 44 h exposure to EZH2i. After treatment, fresh MTT resuspended in phosphate-buffered saline (PBS) was added to each well (final concentration 0.33 mg/ml) and the plate was incubated at 37 °C for 2 h in a humidified incubator with 5% CO_2_. The medium was then removed, and the formazan crystals dissolved in 200 µl of DMSO (Sigma-Aldrich) with gentle agitation. The optical density was measured at 570 nm (Synergy H1 plate reader, BioTek Instruments, Inc., Winooski, VT, USA). Each experiment was performed in six replicates and repeated three times.

### Xenograft experiments

All animal experiments were performed in accordance with the national (Ministero della Salute) and institutional (University of Calabria Animal Welfare Committee; OPBA) guidelines and regulations (protocol n. 170/2024-PR and 139-2017-PR). H295R cells (5 × 10^6^) were inoculated subcutaneously in the interscapular region, and MDA-MB231 cells (1 × 10^6^) were grafted in the mammary fat pad of 8-week-old female Foxn1nu mice (Harlen Envigo). Three weeks later mice were randomly divided into the control group receiving vehicle (20% methyl-β-ciclodextrin, MβC), and treatment groups (TAZ, RSL3, and TAZ + RSL) with 3 (H295R) or 6 (MDA-MB231) mice in each group. TAZ (10 mg/kg), was administered intraperitoneally every-other-day for four weeks, RSL3 (3 mg/kg) used as single agent or in combination with TAZ (TAZ + RSL3; 10 and 3 mg/kg, respectively) was administered intraperitoneally every other day for 2 weeks. The tumor growth was observed and recorded and never exceeded the maximal tumor size set between 1.2 and 1.5 cm in diameter. Tumor volume was calculated using the following equation: volume = length × (width)^2^ × 1/2. Tumors were harvested, weighed, and used for further analyses.

### RNA extraction, reverse transcription, and real-time PCR

Total RNA was extracted using the TRIzol reagent (Invitrogen, Carlsbad, CA). One microgram of total RNA was reverse transcribed into a final volume of 50 μl using the High Capacity cDNA Reverse Transcription Kit (Thermo Fisher, Foster City, CA, USA). cDNA was diluted 1:3 in nuclease-free water and used for real-time PCR. The primer sequences are listed in Table S5. PCR reactions were performed in the QuantStudio^TM^ 3, Real-Time PCR System (Thermo Fisher) using 0.2 μM of each primer. PowerUp™ SYBR™ Green Master Mix (Thermo Fisher) with the dissociation protocol was used for gene amplification; negative controls contained water instead of first-strand cDNA. Each sample was normalized to its 18S rRNA (18S) content. Final results were expressed as *n*-fold differences relative to a calibrator and calculated using the ΔΔCt method. Each experiment was performed in duplicate and repeated three times.

### Lipid staining

Cells were plated into six-well plates containing coverslips at a confluence of 30–50%. After 48 h, cells were treated for 48 h with GSK126 (5 and 25 μM), GSK343 (10 μM), DZNep (5 μM), and Tazemetostat (5 μM). At the end of the treatment, cells were washed two times with PBS and then incubated for 20 min with 10 μM BODIPY™ 493/503 or 10 μM BODIPY™ 558/568 C12. Cells were washed two times with PBS and fixed with 4% PFA for 15 min at room temperature and permeabilized with Triton X-100 (0.2% in PBS) for 3 min. Cells were then washed again and incubated with DAPI solution (0.2 mg/ml in PBS) for 5 min. After a final wash, coverslips were mounted onto glass slides. Fluorescence was visualized using an FV3000 confocal laser scanning microscope (Olympus Corporation, Tokyo, Japan).

### Lipid peroxidation

H295R and MUC-1 cells were plated into six-well plates containing coverslips. After 48 h, cells were treated for an additional 48 h with GSK126 (5 μM). RSL3 was added for 4 h following 44 h exposure to EZH2i. H295R 3D spheroids were grown in 96 wells for 4 days and then incubated for 48 h with appropriate treatments. At the end of the treatment, cells were washed two times with PBS and then incubated for 20 min with 5 μM BODIPY™ C11 581/591. Cells were washed two times with PBS and fixed with 4% PFA for 15 min at room temperature. 3D spheroids were transferred to cavity slides (SIGMA). Coverslips were mounted onto glass slides and fluorescence was visualized using an FV3000 confocal laser scanning microscope (Olympus Corporation, Tokyo, Japan).

### MUFA and PUFA determination

H295R cells (1 × 10^6^ cells) were plated into Petri dishes and treated with GSK126 for 48 h. Cells were then lifted and washed three times by centrifugation in ice-cold PBS prior to lysis. Each cell pellet was subjected to extraction (200 µL of chloroform:isopropanol:NP-40 in a ratio of 7:11:0.1) and centrifugation (10 min at 15,000×*g*). The organic phase was transferred to a new tube, air dried at 50 °C to remove the chloroform, and subjected to vacuum for 30 min to remove the trace amounts of organic solvent. The dried lipids were dissolved in 200 μL of DMSO by a vortex. MUFA and PUFA amount was measured using a colorimetric Lipid Quantification Kit (Cell Biolabs) according to the manufacturer’s instruction.

### LC-MS analysis and data analysis of cell extracts

The LC-MS grade solvents utilized in this study were as follows: LC-MS Chromasolv^TM^ Acetonitrile (ACN) (Riedel-de-Haën^TM^), LC-MS Chromasolv^TM^ Methanol (MeOH) (Riedel-de-Haën^TM^), LiChropur^TM^ Ammonium formate (NH4HCO2) (SIGMA-ALDRICH), and Optima Formic Acid (FAc) (Fisher chemical).

Dried metabolite extracts were obtained from H295R and MDA-MB231 cells (1 × 10^7^) based on the protocol described by Yuan et al. [56] with minor modifications. Briefly, cells were plated in petri dishes for 48 h and then treated with 0, 5, and 25 µM GSK126. After 48 h, the medium was removed and washed with 10 ml of NaCl 0,9%. Then, cells were collected in an extraction solution, MeOH:water:ACN (5:3:1) cooled to −80 °C, allowed to obtain a good extraction yield of long aliphatic chain polar metabolites. Dried pellets were dissolved in organic solution ACN:MeOH:water (70:10:20), adding internal standards for quality control. Resuspended samples were centrifuged at 4 °C for 10 min at 15,000 RCF before MS analysis. The quality control (QC) sample consisted of a mixture of equal volumes of each sample. Analysis of samples was carried out using a Vanquish^TM^ Flex UHPLC system (Thermo Scientific, Waltham, USA) coupled to an Orbitrap Fusion^TM^ Tribrid™ mass spectrometer (Thermo Fisher Scientific) equipped with a heated electrospray ion (HESI) source.

Samples were analyzed in a randomized way, bracketed by a blank and pooled QC sample for background correction and normalization of the data, respectively. Two internal standards were used, 3-acetyl-indole and *N*-acetyl-l-phenylalanine (5 ppm). The injection volume was 2.0 μL. Metabolites were separated on a Thermo Fisher Accucore^TM^ -150-Amide-HILIC column (100 × 2.1 mm; 2.6 µm) with a flow rate of 300 µL min^−1^ (column temperature 30 °C). For HILIC separation (Eluents: A: 95% ACN, 5% H_2_O, 10 mM NH_4_HCO_2_, 0.1% FAc, B: 30% ACN, 70% H_2_O, 10 mM NH_4_HCO_2_, 0.1% FAc) the following gradient was used: 0.0–1.0 min 1% B, 1.0–12.0 min gradual increase to 90% B, 12.0–14.0 min 90% B, and at 14.5 min switch to 1% B and re-equilibration until 21 min. The operating conditions of the HESI source were: ionization potential +3.5/−3.3 kV, ion transfer tube temperature at 300 °C, vaporizer temperature at 300 °C, sheath gas flow to 40, and auxiliary gas flow to 8. Mass spectrometry analyses were performed in positive and negative ion mode with a scan range of m/z 50 to 1000 at a mass resolution of 120,000 for full-scan MS. MS/MS spectra were acquired by data-dependent mode at 60,000 mass resolution with two different collision modes: HCD (for MS2) at collision energies of 20, 40, and 90 a.u.; CID (for MS3) at collision energies of 30%. Blank and noise signals were subtracted using AcquireX^TM^ software from Xcalibur^TM^ 4.2 Thermo Scientific^TM^ from raw data files. Obtained data sets were processed by Compound Discoverer^TM^ 3.3.0.5 (Thermo Fisher Scientific, Bremen, Germany), using a workflow for untargeted metabolomics analysis. The parameters used in the analysis workflow were: mass tolerance 5 ppm, retention time tolerance 0.2 min, area calculation and normalization minimum peak 100,000 a.u. of intensity. Compounds were annotated by comparing the retention time and MSn match against our internal mass list database, by using the mzVault node in the data processing software and through the Fragment Ion Search (FISh) scoring tool. Several databases present in the cloud were queried: Human Metabolome Database, BioCyc, ChEBI, ChEMBL, LipidMAPS. The putative identification of annotated compounds corresponded to level B1 or B2 [57].

Data represent the analysis of three biological replicates for control cells and three replicates for GSK126-treated samples normalized to the cell number.

Metabolite enrichment analysis and Joint pathway analysis was performed using MetaboAnalyst 6.0 (https://www.metaboanalyst.ca/) (accessed on 16 February 2024) [58, 59].

Specifically, the metabolite enrichment analysis was done by uploading a list of compounds to assess the pathway enrichment of metabolites significantly modulated in H295R cells, obtaining a dot plot as a result. The size of the circles for each metabolite set represents the enrichment ratio, while the color indicates the *p* value.

The joint pathway analysis was conducted to provide an overview of the pathway enrichment analysis using significantly different transcripts (gene list) and metabolites (compound list) in GSK126-treated cells, obtaining a scatter plot.

The x-axis shows pathway impact scores, which summarize normalized topology measures of those perturbed genes/metabolites in each pathway. The y-axis shows −log10(*P*) values of the enrichment analysis results. The sizes of the data points are correlated with their x values, and the color gradients correspond to their y values [59].

Pathway impact is calculated as the sum of the importance measures of matched metabolites normalized by the sum of the importance measures of all metabolites in each pathway [60].

The significant pathways were selected based on the criteria of the log10(raw *P*) > 2.

### Determination of oxidative stress

Oxidative stress was evaluated using the Muse Oxidative Stress Kit (Luminex). H295R and MUC-1 cells were plated in 6-well plates containing growth medium, and then treated for 48 h with 5 and 25 µM GSK126. Cells were trypsinized, counted, and 1 × 10^6^ cells resuspended in 1 ml of the reagent 1X Assay Buffer. In a test tube, 10 µl of cells (~1 × 10^4^) from each sample were added to 190 µl of Muse Oxidative Stress Reagent (working solution) and incubated for 30 min at 37 °C. Guava® Muse® Cell Analyzer - Luminex instrument was used to analyze 5000 events. Positive control was obtained by treating cells with H_2_O_2_ (3 mM final concentration) for 2 h. Each experiment was performed in six replicates and repeated three times. Analyses were performed on six samples per condition.

### Glutathione determination

Total glutathione (GSH) content was evaluated using the Glutathione Colorimetric Detection Kit (Invitrogen). H295R cells were plated in six-well plates containing growth medium, and treated for 48 h with 5 and 25 µM GSK126. Cells were trypsinized and resuspended in 1 ml of ice-cold 5% aqueous solution of 5-sulfo-salicylic acid dihydrate (SSA) (1 × 10^6^). Cells were lysed by vigorous vortexing, incubated for 10 min at 4 °C, and centrifuged at 14,000 rpm for 10 min at 4 °C. Supernatants were diluted by adding 4X v/v of 1X Assay Buffer. Cell extracts were then incubated with the Reaction Mixture for 20 min and used to detect total GSH according to the manufacturer’s instructions. Absorbance was measured at 405 nm (Synergy H1 plate reader, BioTek Instruments, Inc., Winooski, VT, USA). Each experiment was performed in six replicates and repeated three times.

### Patients’ databases analysis

Bioinformatics analyses from transcriptome data were performed in R (http://www.R-project.org) using software packages from the Bioconductor portal (www.bioconductor.org). To compare gene expression levels between normal adrenal cortex, ACA, and ACC, we downloaded microarray data (.CEL files) from GEO (series GSE33371) [61]. To import microarray expression data into R, to summarize probe-level expression values, and to perform gcrma normalization, we used R/Bioconductor packages affy [62] and gcrma: https://bioconductor.org/packages/gcrma. To remove duplicated probes and ENTREZ ids, we used the feature Filter function from genefilter, R package version 1.82: 10.18129/B9.bioc.genefilter. We used the ggboxplot and stat_compare_means functions from ggpubr (https://CRAN.R-project.org/package=ggpubr) to build the boxplots, and to assess statistical significance among the classes. To assess the effect of gene expression on survival, we used transcriptome data from the ACC-TCGA dataset [63]. We downloaded legacy RNA-seq counts data from the Genomic Data Commons (GDC) portal using TCGA biolinks [64] and performed log2-cpm normalization using edgeR, after correcting for library size using the TMM method [65]. For overall survival analysis, we split the ACC-TCGA cohort into two groups according to the median expression of each gene. We used Kaplan–Meier plots to depict survival times, and the log-rank test to assess statistical significance.

To evaluate the combined effects of EZH2 expression and ferroptosis genes on survival, we stratified the cohort into four groups based on the median expression of EZH2 and each ferroptosis gene. Kaplan–Meier plots were utilized to illustrate survival times, and the log-rank test was employed to determine statistical significance. Furthermore, we applied Cox regression models to estimate the impact of EZH2, both independently, and in combination with other ferroptosis genes, on overall survival. To assess whether incorporating a second variable (ferroptosis genes) into the univariate model with EZH2 improved the model’s fit, we performed likelihood-ratio tests.

### Transmission electron microscopy (TEM) analysis

Immediately after collection, cell pellets were fixed in 3% glutaraldehyde solution in 0.1 M phosphate buffer (pH 7.4) for 2 h at 4 °C. After osmium tetroxide post-fixation and buffer washes, samples were dehydrated through acetone-graded series and then progressively embedded in acetone/resin with a final embedment in pure resin (Araldite-Fluka). Afterward, the samples were transferred to a fresh resin mixture in embedding capsules and polymerized in an oven at 60 °C for about 72 h. Ultrathin sections were cut with a diamond knife, mounted on copper grids (G300 Cu), and imaged using a Jeol JEM 1400-Plus electron microscope operating at 80 kV.

### Statistical analysis

All experiments were performed at least three times. Data are expressed as mean values ± standard error (SE). The statistical significance was analyzed using GraphPad Prism 5.0 software (GraphPad Software, Inc., San Diego, CA, USA). Groups were compared using the analysis of variance (ANOVA) with Bonferroni’s post hoc testing. Significance was defined as *p* < 0.05.

## Supplementary information

Supplementary figures S1-7

Supplementary tables S1-5

Dataset1

Dataset2

Original Data

## Data Availability

All data generated during this study are included in this published article and its supplementary information files.
