# Supplementary material for: A targetable antioxidant defense mechanism to EZH2 inhibitors enhances tumor cell vulnerability to ferroptosis
Source: Cell Death Dis. 2025 Apr 14;16(1):291. doi: 10.1038/s41419-025-07607-y (PMC11997205; doi:10.1038/s41419-025-07607-y)
Supplement: Supplementary file 1 — Supplementary figures S1-7 [file 41419_2025_7607_MOESM1_ESM.pdf]

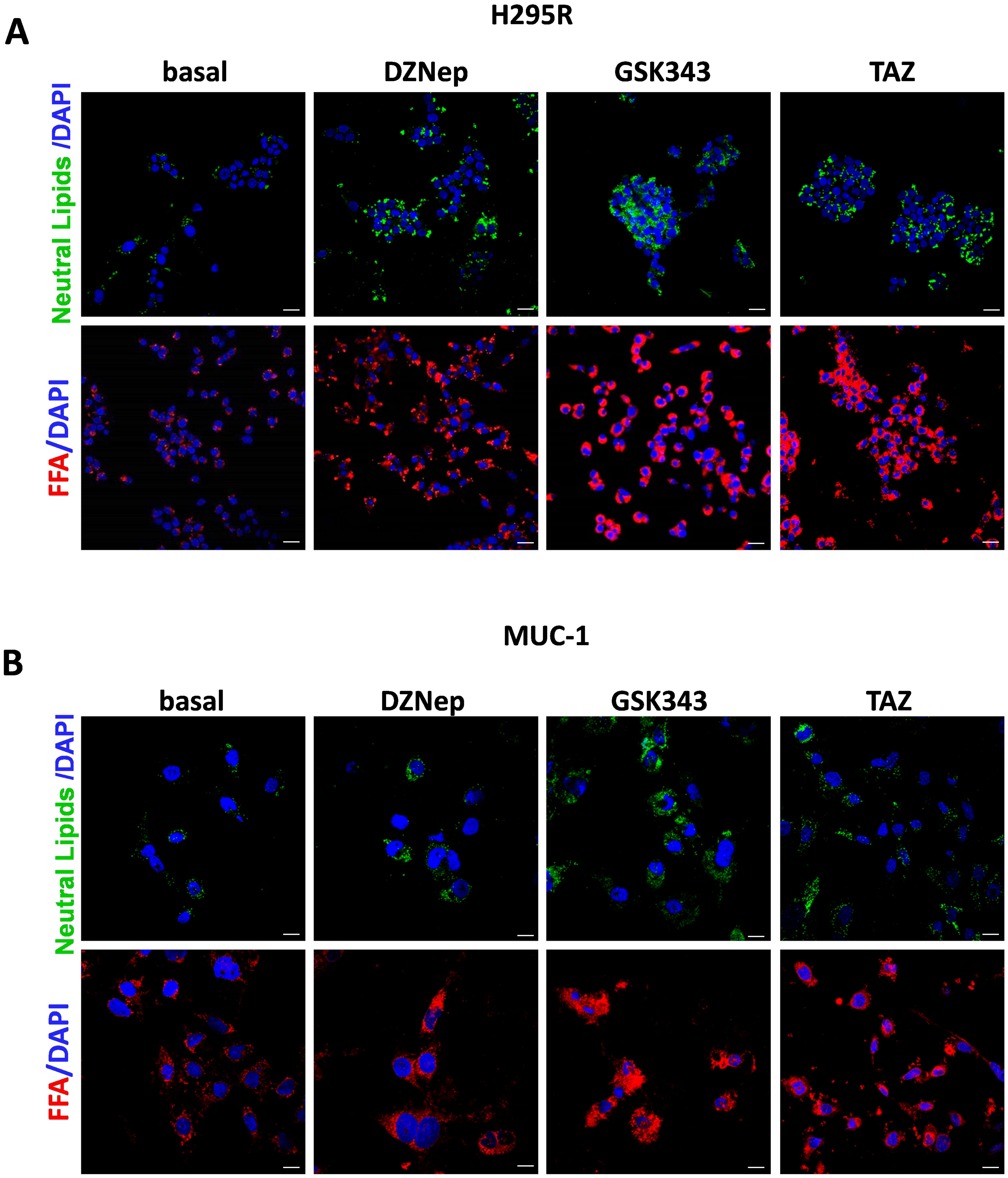

Figure S1. EZH2i affect lipid content in ACC cells.
A, B. Confocal images of LDs stained by BODIPY 493/503 fluorescent dye (Neutral lipids) and BODIPY™ 558/568 C12 (free fatty acids, FFA) in H295R **(A)** and MUC-1 **(B)** cells treated for 48h with DZNep (5 μM), GSK343 (10 μM) and Tazemetostat (TAZ, 5 μM). Nuclei were stained by DAPI. (scale bar 50 μm)

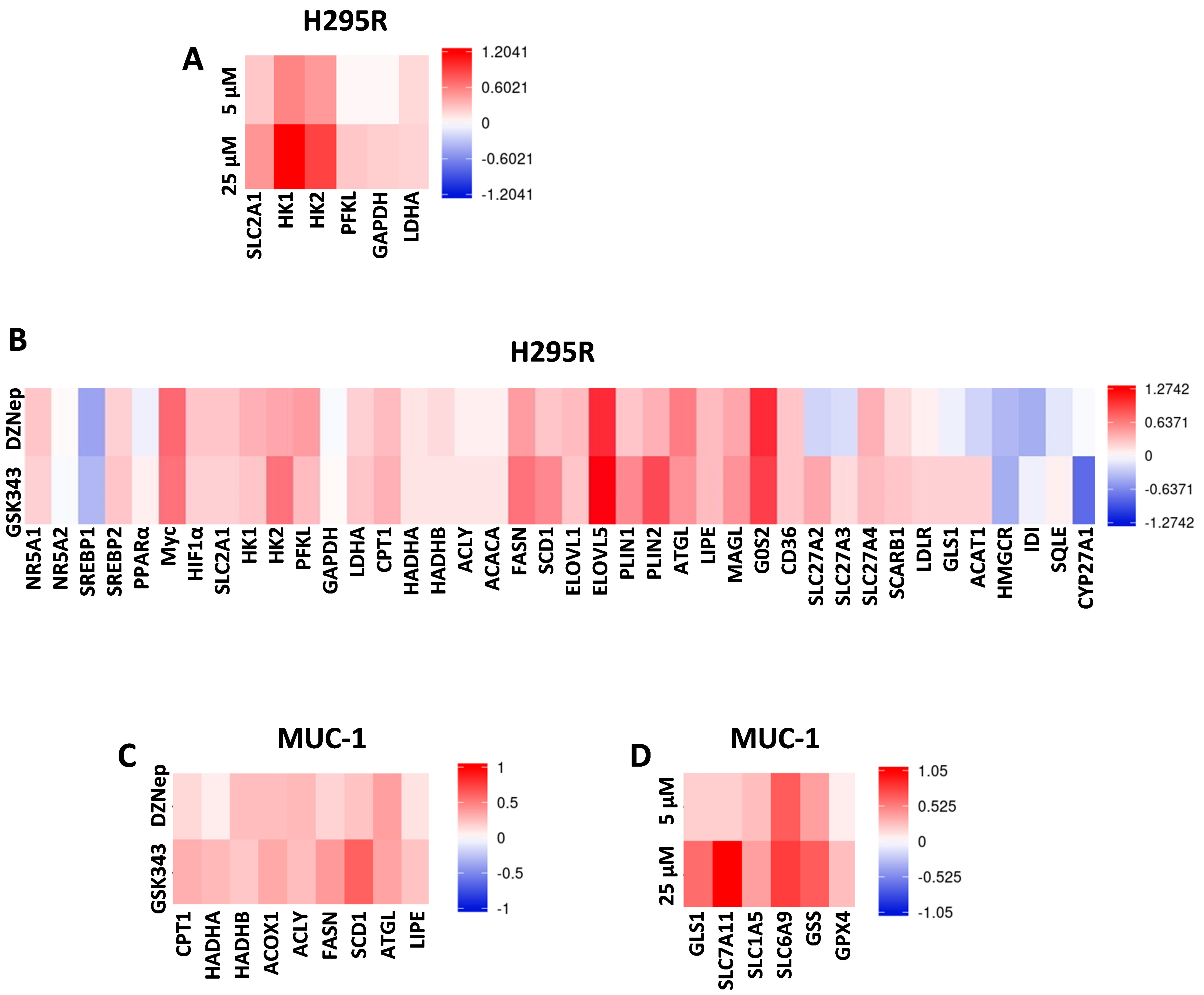

Figure S2. Effects of EZH2i on the expression of metabolic genes.
A-D. Heatmaps of mRNA expression of metabolic genes in ACC cells evaluated by real-time RT-PCR after 48 h treatment with GSK126 (5 and 25 µM) (**A, D**), DZNep (5 µM) and GSK343 (10 µM) (**B, C**). Data are the values from 3 separate RNA samples presented as log10 of fold-change.

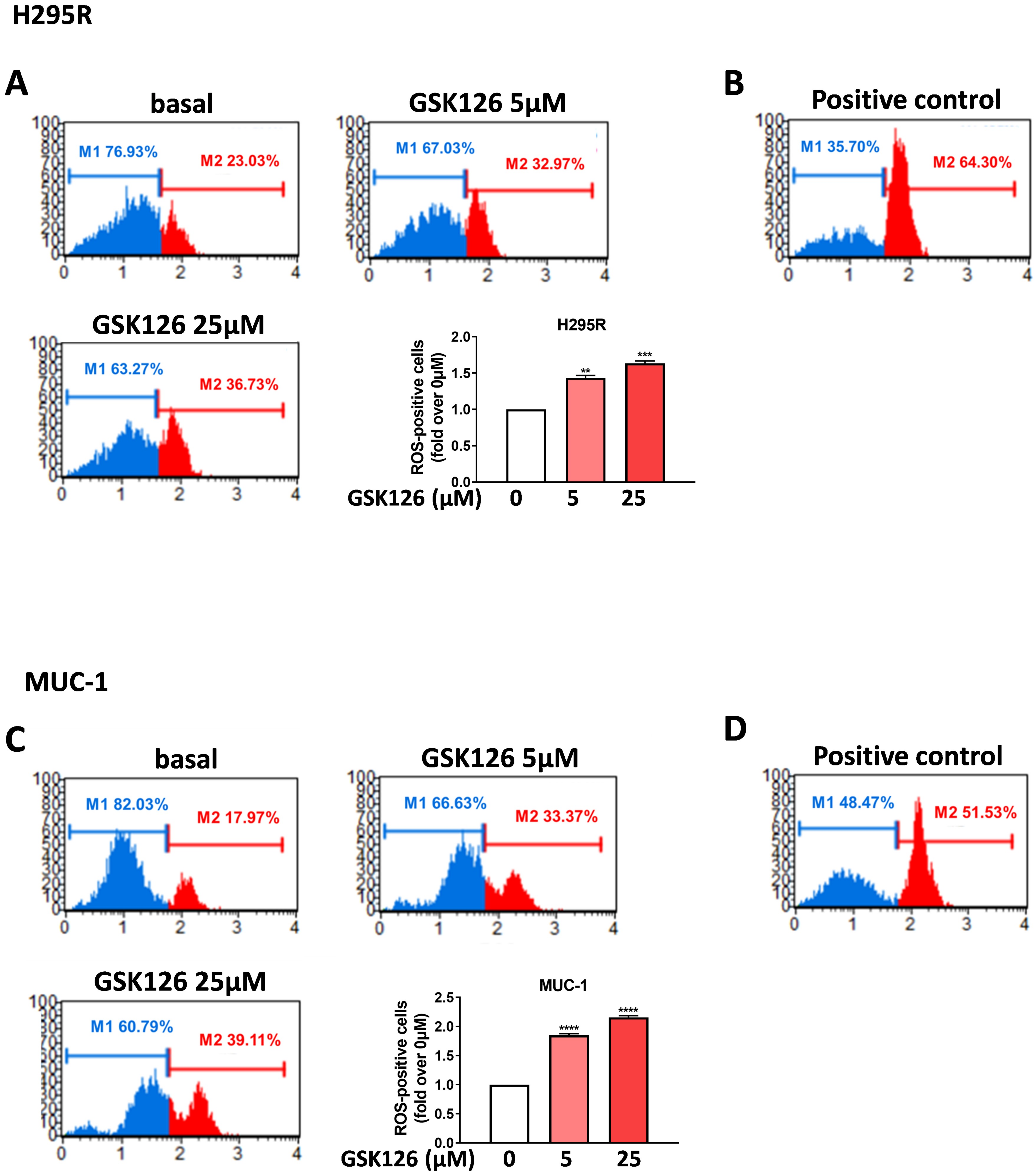

Figure S3. EZH2i increase ROS in ACC cells.

A-D. Flow cytometry analysis of reactive oxygen species (ROS) in ACC cells treated with GSK126 for 48 h. Cells treated with H_2O_2 for 2 h were used as positive control (**B, D**). ROS-positive cells are shown in red. Histograms represent the fold change in the number of ROS-positive cells. Data are expressed as mean ± SEM. n=3 independent experiments. ** p <0.01, *** p <0.001, **** p <0.0001.

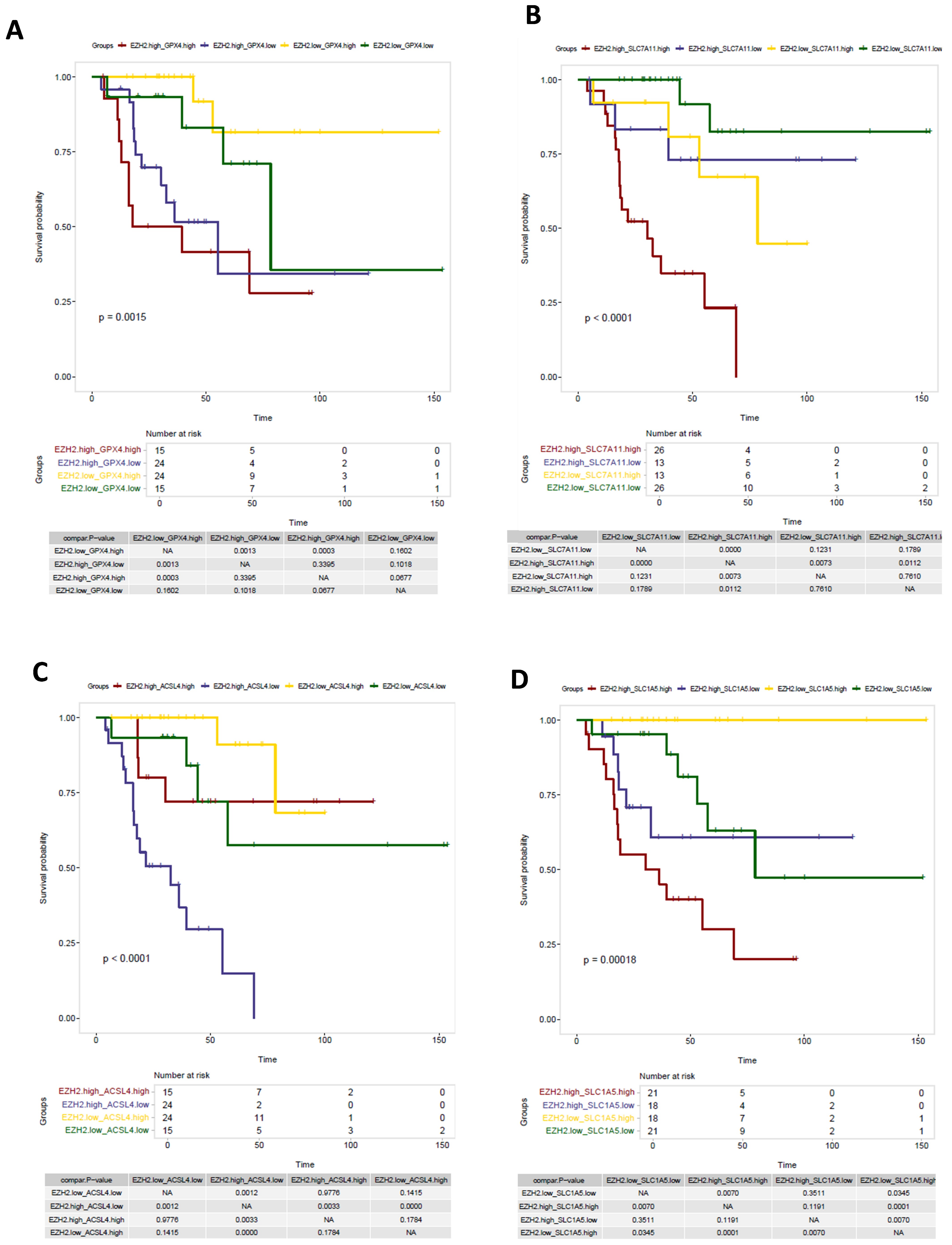
A
Groups EZH2.high_GPX4.high EZH2.high_GPX4.low EZH2.low_GPX4.high EZH2.low_GPX4.low
Survival probability
p = 0.0015
Time
Number at risk
EZH2.high_GPX4.high 15 5 0 0
EZH2.high_GPX4.low 24 4 2 0
EZH2.low_GPX4.high 24 9 3 1
EZH2.low_GPX4.low 15 7 1 1
compar.P−value EZH2.low_GPX4.high EZH2.high_GPX4.low EZH2.high_GPX4.high EZH2.low_GPX4.low
EZH2.low_GPX4.high NA 0.0013 0.0003 0.1602
EZH2.high_GPX4.low 0.0013 NA 0.3395 0.1018
EZH2.high_GPX4.high 0.0003 0.3395 NA 0.0677
EZH2.low_GPX4.low 0.1602 0.1018 0.0677 NA
B
Groups EZH2.high_SLC7A11.high EZH2.high_SLC7A11.low EZH2.low_SLC7A11.high EZH2.low_SLC7A11.low
Survival probability
p < 0.0001
Time
Number at risk
EZH2.high_SLC7A11.high 26 4 0 0
EZH2.high_SLC7A11.low 13 5 2 0
EZH2.low_SLC7A11.high 13 6 1 0
EZH2.low_SLC7A11.low 26 10 3 2
compar.P−value EZH2.low_SLC7A11.low EZH2.high_SLC7A11.high EZH2.low_SLC7A11.high EZH2.high_SLC7A11.l
EZH2.low_SLC7A11.low NA 0.0000 0.1231 0.1789
EZH2.high_SLC7A11.high 0.0000 NA 0.0073 0.0112
EZH2.low_SLC7A11.high 0.1231 0.0073 NA 0.7610
EZH2.high_SLC7A11.low 0.1789 0.0112 0.7610 NA
C
Groups EZH2.high_ACSL4.high EZH2.high_ACSL4.low EZH2.low_ACSL4.high EZH2.low_ACSL4.low
Survival probability
p < 0.0001
Time
Number at risk
EZH2.high_ACSL4.high 15 7 2 0
EZH2.high_ACSL4.low 24 2 0 0
EZH2.low_ACSL4.high 24 11 1 0
EZH2.low_ACSL4.low 15 5 3 2
compar.P−value EZH2.low_ACSL4.low EZH2.high_ACSL4.low EZH2.high_ACSL4.high EZH2.low_ACSL4.high
EZH2.low_ACSL4.low NA 0.0012 0.9776 0.1415
EZH2.high_ACSL4.low 0.0012 NA 0.0033 0.0000
EZH2.high_ACSL4.high 0.9776 0.0033 NA 0.1784
EZH2.low_ACSL4.high 0.1415 0.0000 0.1784 NA
D
Groups EZH2.high_SLC1A5.high EZH2.high_SLC1A5.low EZH2.low_SLC1A5.high EZH2.low_SLC1A5.low
Survival probability
p = 0.00018
Time
Number at risk
EZH2.high_SLC1A5.high 21 5 0 0
EZH2.high_SLC1A5.low 18 4 2 0
EZH2.low_SLC1A5.high 18 7 2 1
EZH2.low_SLC1A5.low 21 9 2 1
compar.P−value EZH2.low_SLC1A5.low EZH2.high_SLC1A5.high EZH2.high_SLC1A5.low EZH2.low_SLC1A5.high
EZH2.low_SLC1A5.low NA 0.0070 0.3511 0.0345
EZH2.high_SLC1A5.high 0.0070 NA 0.1191 0.0001
EZH2.high_SLC1A5.low 0.3511 0.1191 NA 0.0070
EZH2.low_SLC1A5.high 0.0345 0.0001 0.0070 NA

E

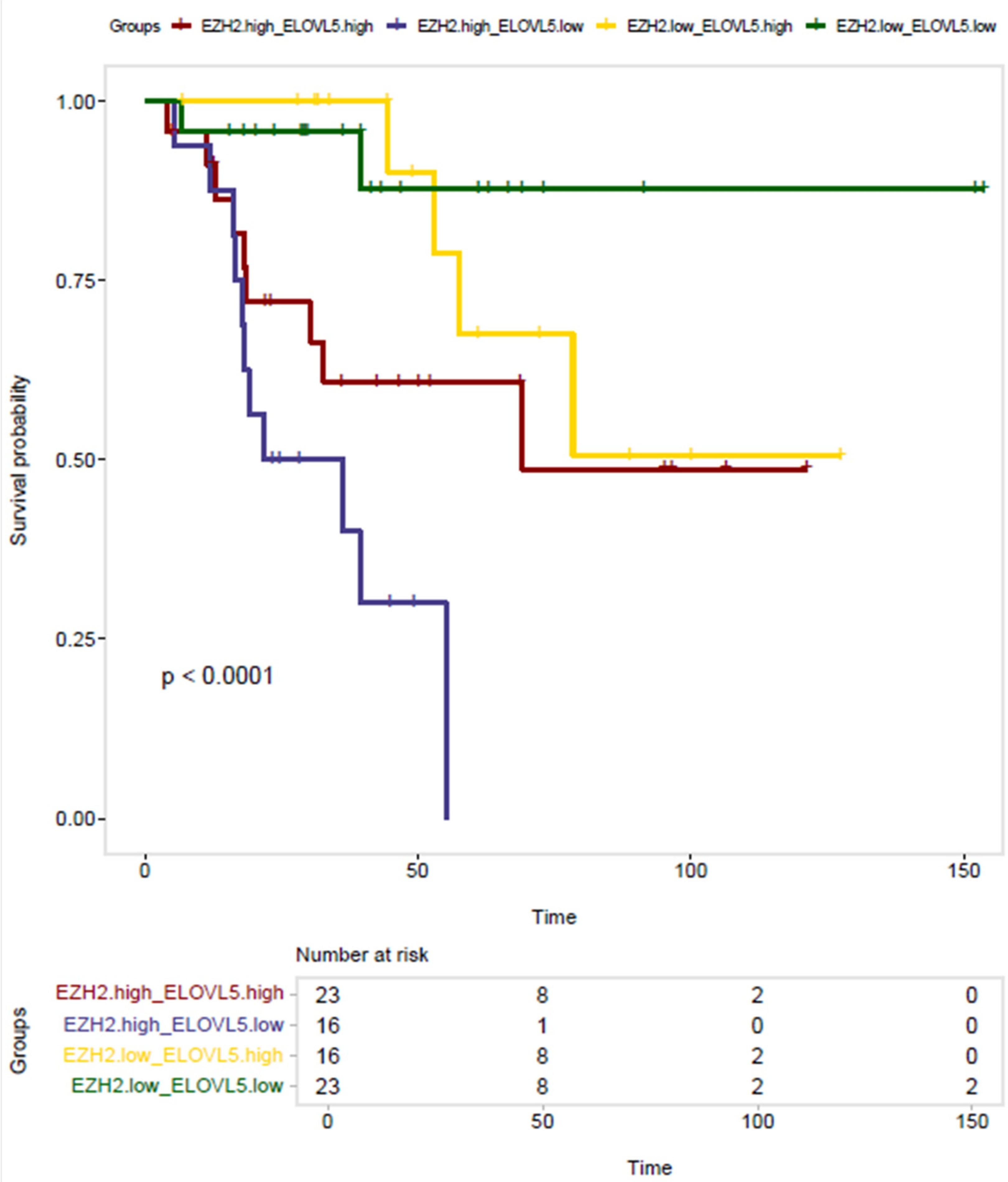

compar.P−value	EZH2.low_ELOVL5.high	EZH2.high_ELOVL5.low	EZH2.high_ELOVL5.high	EZH2.low_ELOVL5.low
EZH2.low_ELOVL5.high	NA	0.0001	0.1942	0.3901
EZH2.high_ELOVL5.low	0.0001	NA	0.0601	0.0000
EZH2.high_ELOVL5.high	0.1942	0.0601	NA	0.0233
EZH2.low_ELOVL5.low	0.3901	0.0000	0.0233	NA

F

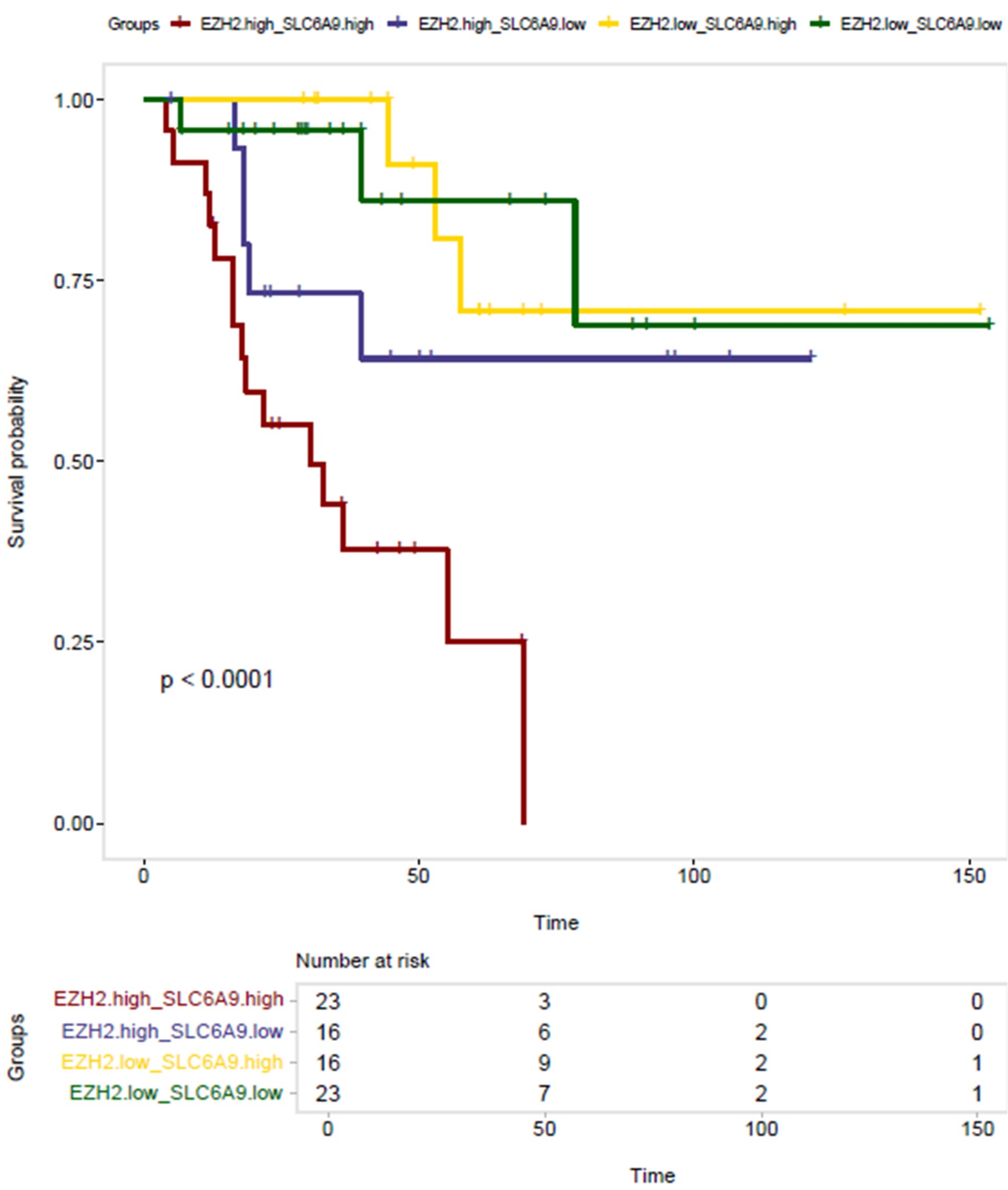

compar.P−value	EZH2.low_SLC6A9.high	EZH2.high_SLC6A9.high	EZH2.low_SLC6A9.low	EZH2.high_SLC6A9.low
EZH2.low_SLC6A9.high	NA	0.0003	0.9573	0.2012
EZH2.high_SLC6A9.high	0.0003	NA	0.0001	0.0289
EZH2.low_SLC6A9.low	0.9573	0.0001	NA	0.2272
EZH2.high_SLC6A9.low	0.2012	0.0289	0.2272	NA

Figure S4. Risk stratification according to the expression of EZH2 and ferroptosis-related genes in ACC patients. A-F. Kaplan-Meier curves showing overall survival (in months) of subgroups of patients stratified according to the median expressions of EZH2 and other ferroptosis-related genes. The total number of patients in each subgroup is shown in the middle panel. The p-value on top of the plot represents the overall result of the log-rank test including all groups. Tables at the bottom panels of each figure indicate the p-values of pairwise log-rank tests between each subgroup.

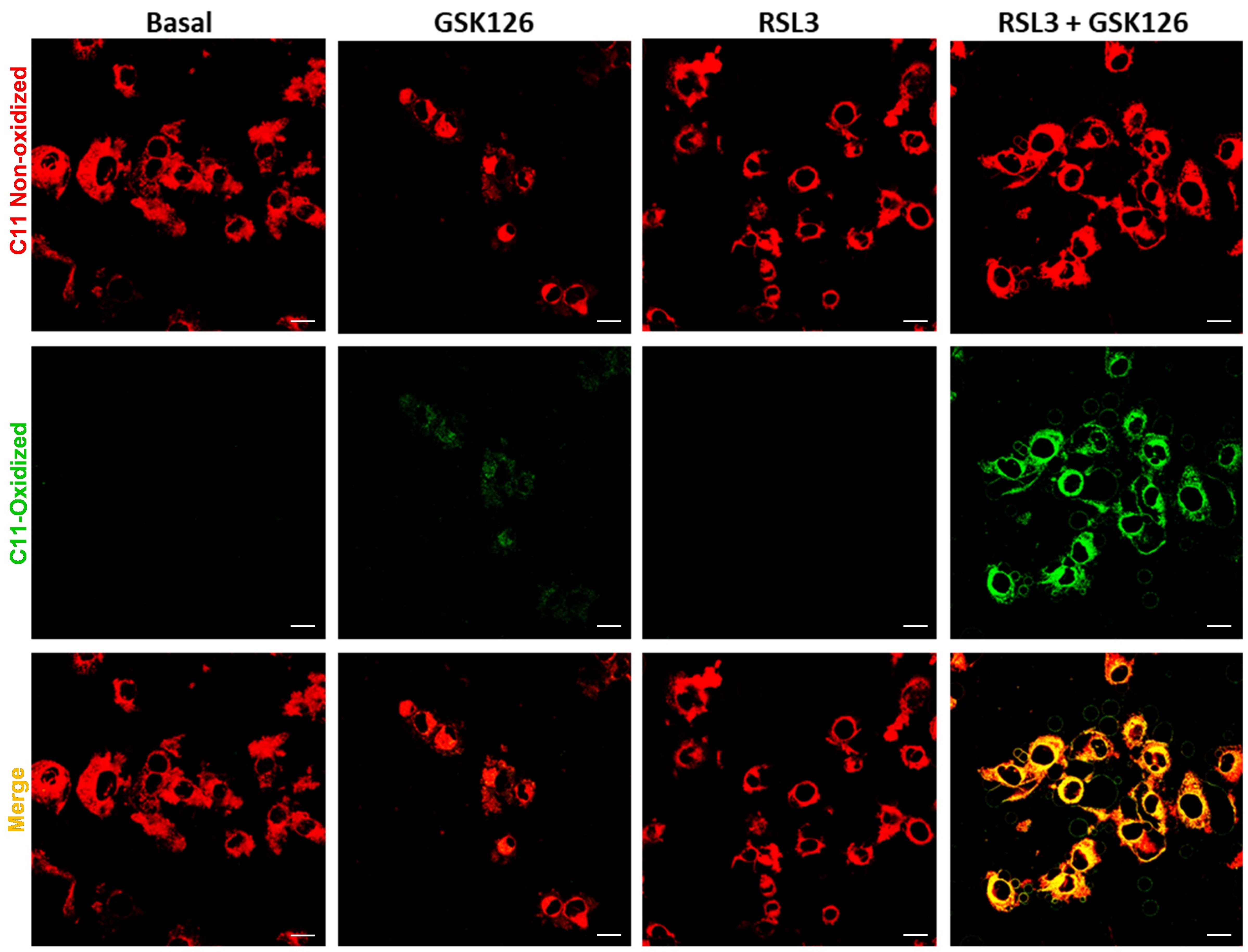

Figure S5. EZH2i sensitize MUC-1 cells to ferroptosis inducers.
A. Confocal images of lipid peroxidation detected by BODIPY C11 fluorescent dye in MUC-1 cells treated for 48 h with GSK126 (5 μM) in the presence or absence of RSL3 (1 μM, for the last 4h) (scale bar 50 μm).

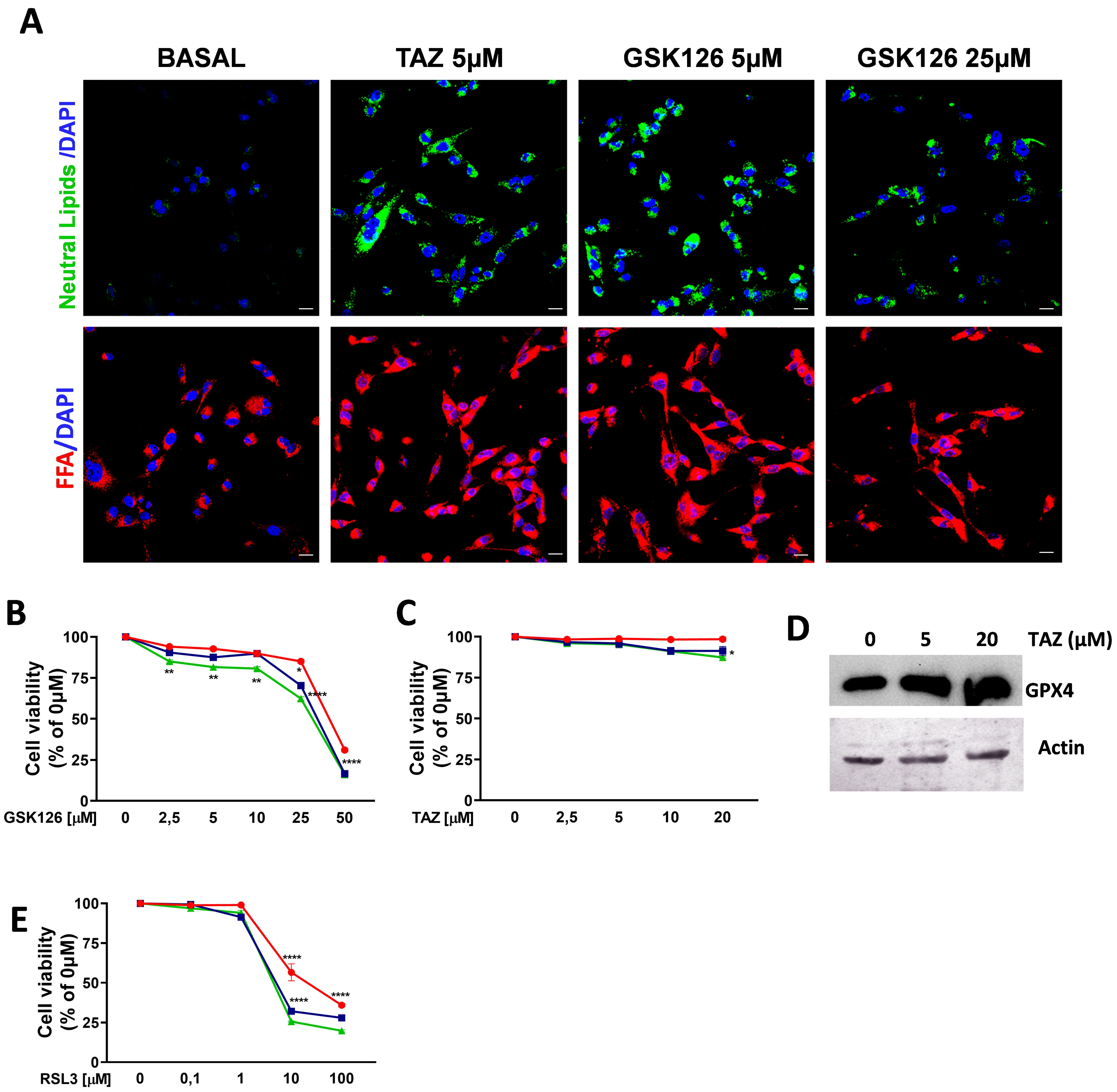

Figure S6. EZH2i increase lipid content and GPX4 expression in a cell model of TNBC. **A.** Confocal images of lipid droplets stained by BODIPY 493/503 fluorescent dye (Neutral lipids) and BODIPY™ 558/568 C12 (free fatty acids, FFA) in MDA-MB231 cells treated for 48 h with TAZ (5 µM) and GSK126 (5 µM and 25 µM). Nuclei were stained by DAPI. Scale bar 50 µm. **B, C, E.** Cell viability by MTT assay of MDA-MB231 cells treated for 24, 48 and 72 h with the indicated doses of GSK126, TAZ and RSL3. n=3 independent experiments. **D.** Western blot of GPX4 in MDA-MB231 after 48 h of TAZ treatment. Actin was used as a loading control. Data are expressed as means ± SEM. * $p < 0.05$; ** $p < 0.01$ **** $p < 0.0001$.

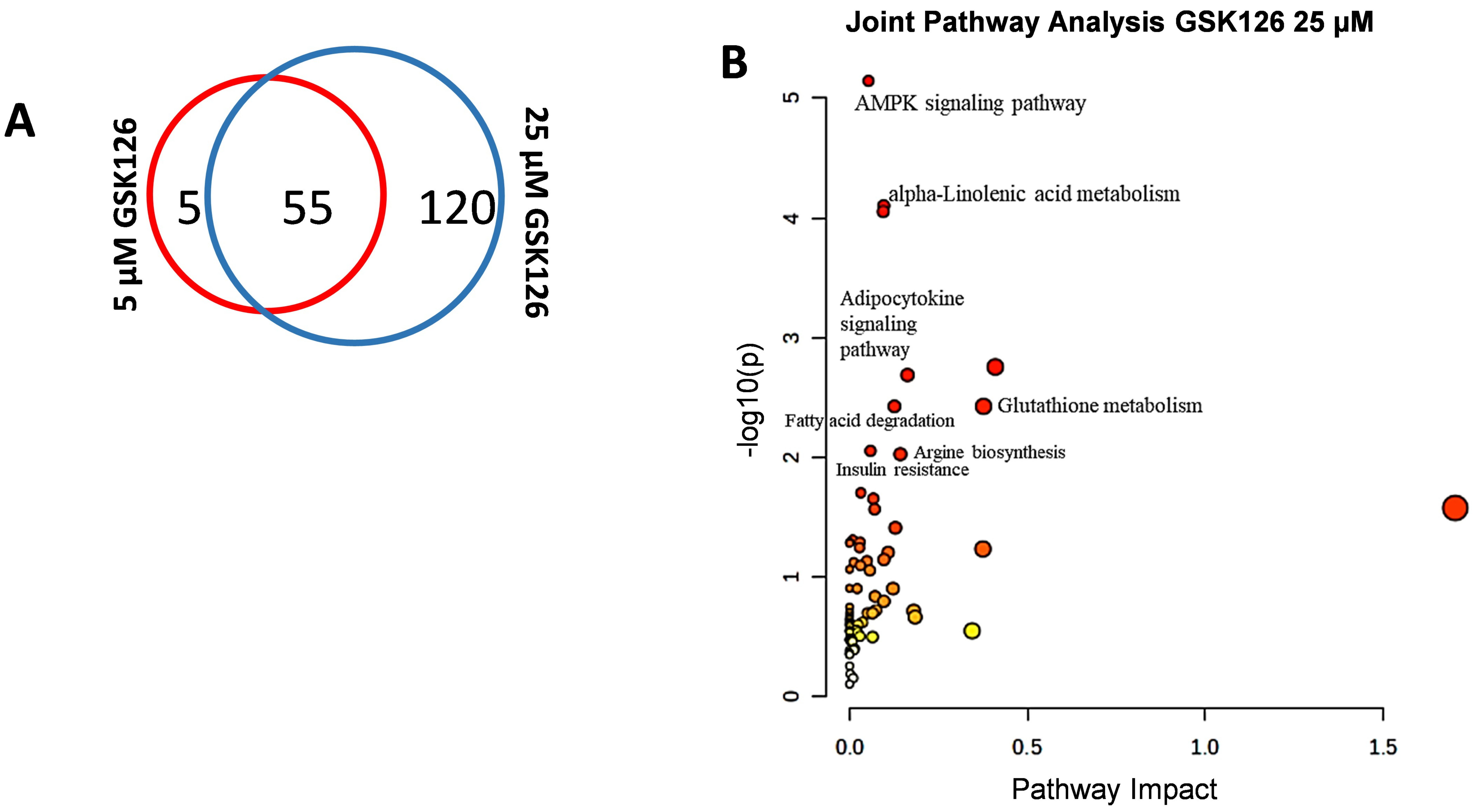

Figure S7. GSK126-treated TNBC cells are enriched in lipid and antioxidant genes and metabolites.

A. Untargeted metabolomics analysis was performed on MDA-MB231 cells treated with GSK126 (5 µM and 25 µM) for 48 h. Venn diagram represents statistically significant differentially abundant metabolites across 5 and 25 µM GSK126-treated cells. **B.** Overview of the pathway enrichment analysis based on both transcripts and metabolites with statistically significant difference in 25 µM GSK126-treated MDA-MB231 cells. Only pathways with –log10(p) > 2 are shown.
