# Supplementary material for: A targetable antioxidant defense mechanism to EZH2 inhibitors enhances tumor cell vulnerability to ferroptosis
Source: Cell Death Dis. 2025 Apr 14;16(1):291. doi: 10.1038/s41419-025-07607-y (PMC11997205; doi:10.1038/s41419-025-07607-y)
Supplement: Supplementary file 2 — Supplementary tables S1-5 [file 41419_2025_7607_MOESM2_ESM.docx]

**Tables**

**Table S1**: Joint Pathway Analysis of 5 μM GSK126-treated H295R cells

| **Pathways** | **Total** | **Hits** | **Raw p** | **-LOG10(p)** | **FDR** | **Impact** |
| --- | --- | --- | --- | --- | --- | --- |
| Insulin resistance | 128 | 8 | 3.75E-07 | 6.4257 | 6.21E-05 | 0.095588 |
| Fatty acid degradation | 94 | 7 | 6.47E-07 | 6.1891 | 7.14E-05 | 2.1681 |
| Regulation of lipolysis in adipocytes | 69 | 5 | 0.0000328 | 4.4843 | 0.002713 | 0.15 |
| Adipocytokine signaling pathway | 76 | 5 | 0.0000524 | 4.2809 | 0.003467 | 0.27907 |
| Central carbon metabolism in cancer | 106 | 6 | 0.00025398 | 3.5952 | 0.01201 | 0.19469 |
| beta-Alanine metabolism | 63 | 4 | 0.00035514 | 3.4496 | 0.014503 | 0.3333 |
| Butanoate metabolism | 70 | 4 | 0.00053156 | 3.2744 | 0.015476 | 0.29091 |
| Arginine biosynthesis | 44 | 4 | 0.0016733 | 2.7764 | 0.042604 | 0.17143 |
| Lysine degradation | 115 | 4 | 0.0033491 | 2.4751 | 0.069284 | 0.090909 |
| Alanine aspartate and glutamate metabolism | 64 | 3 | 0.0048783 | 2.3117 | 0.089706 | 0.39344 |
| alpha-Linolenic acid metabolism | 69 | 3 | 0.0060193 | 2.2205 | 0.10369 | 0.23077 |
| Pyruvate metabolism | 70 | 3 | 0.006265 | 2.2031 | 0.10369 | 0.15094 |

**Table S2.** Joint Pathway Analysis of 25 μM GSK126-treated H295R cells

| **Pathways** | **Total** | **Hits** | **Raw p** | **-LOG10(p)** | **FDR** | **Impact** |
| --- | --- | --- | --- | --- | --- | --- |
| Fatty acid degradation | 94 | 8 | 6,35E-08 | 7.1974 | 1.05E-05 | 2.2017 |
| Insulin resistance | 128 | 8 | 6,98E-07 | 6.1561 | 0.000077 | 0.095588 |
| Regulation of lipolysis in adipocytes | 69 | 5 | 4,81E-05 | 4.3178 | 0.003657 | 0.15 |
| Ferroptosis | 71 | 5 | 5,52E-05 | 4.2577 | 0.003657 | 0.094595 |
| Adipocytokine signaling pathway | 76 | 5 | 7,67E-05 | 4.1155 | 0.004229 | 0.27907 |
| Glutathione metabolism | 94 | 4 | 0,00215 | 2.6676 | 0.064686 | 0.49275 |
| Cysteine and methionine metabolism | 112 | 4 | 0,00405 | 2.3926 | 0.095752 | 0.048544 |
| Lysine degradation | 115 | 4 | 0,00445 | 2.3516 | 0.098196 | 0.090909 |
| beta-Alanine metabolism | 63 | 3 | 0,005819 | 2.2352 | 0.1133 | 0.25926 |
| alpha-Linolenic acid metabolism | 69 | 3 | 0,00749 | 2.1255 | 0.13577 | 0.23077 |
| Butanoate metabolism | 70 | 3 | 0,007793 | 2.1083 | 0.13577 | 0.29091 |

**Table S3.** Summary results of bivariate Cox regression models including the expression of EZH2 and candidate genes in each model. evaluating the effect of the addition of a second variable on overall survival (HR: Hazard ratio; LRT_P: likelihood-ratio test p-value).

| **Model** | **Variable** | **Coefficient** | **HR** | **PValue** | **LRT_PValue** |
| --- | --- | --- | --- | --- | --- |
| **EZH2_GPX4** | **EZH2** | **1.06697336** | **2.9065690** | **8.624824e−07** | **7.224228e−01** |
| **EZH2_GPX4** | **GPX4** | **−0.09018759** | **0.9137598** | **7.208006e−01** | **−** |
| **EZH2_SLC7A11** | **EZH2** | **0.85067786** | **2.3412333** | **8.256629e−05** | **3.070264e−03** |
| **EZH2_SLC7A11** | **SLC7A11** | **0.33857233** | **1.4029432** | **5.004133e−03** | **−** |
| **EZH2_SLC1A5** | **EZH2** | **1.10338647** | **3.0143568** | **1.396681e−06** | **7.201768e−01** |
| **EZH2_SLC1A5** | **SLC1A5** | **−0.04482066** | **0.9561689** | **7.217437e−01** | **−** |
| **EZH2_SLC6A9** | **EZH2** | **1.03992767** | **2.8290124** | **1.824795e−06** | **4.509193e−03** |
| **EZH2_SLC6A9** | **SLC6A9** | **0.35657081** | **1.4284227** | **7.638006e−03** | **−** |
| **EZH2_ACSL4** | **EZH2** | **1.13286498** | **3.1045382** | **1.861178e−06** | **2.868303e−04** |
| **EZH2_ACSL4** | **ACSL4** | **−0.61165479** | **0.5424525** | **1.237762e−04** | **−** |
| **EZH2_ELOVL5** | **EZH2** | **1.12311769** | **3.0744244** | **2.222846e−07** | **2.880245e−01** |
| **EZH2_ELOVL5** | **ELOVL5** | **−0.27510496** | **0.7594924** | **2.849914e−01** | **−** |

**Table S4.** Joint Pathway Analysis of 25 μM GSK126-treated MDA-MB-231 cells

| **Pathways** | **Total** | **Hits** | **Raw p** | **-LOG10(p)** | **FDR** | **Impact** |
| --- | --- | --- | --- | --- | --- | --- |
| AMPK signaling pathway | 143 | 6 | 7.14E-06 | 5.1464 | 0.001182 | 0.053191 |
| alpha-Linolenic acid metabolism | 69 | 4 | 7.91E-05 | 4.102 | 0.007318 | 0.096154 |
| Adipocytokine signaling pathway | 76 | 3 | 0.002058 | 2.6866 | 0.11352 | 0.16279 |
| Fatty acid degradation | 94 | 3 | 0.003765 | 2.4243 | 0.15576 | 0.12605 |
| Glutathione metabolism | 94 | 3 | 0.003765 | 2.4243 | 0.15576 | 0.37681 |
| Insulin resistance | 128 | 3 | 0.008868 | 2.0522 | 0.31292 | 0.058824 |
| Arginine biosynthesis | 44 | 2 | 0.009454 | 2.0244 | 0.31292 | 0.14286 |

**Table S5:** Human primer sequences for real-time PCR

| **Gene name** | **Forward primers 5’- 3’** | **Reverse primers 5’- 3’** |
| --- | --- | --- |
| 18S | CGGCGACGACCCATTCGAAC | GAATCGAACCCTGATTCCCCGTC |
| ACACA | CAGGTCACACGTCTCTTTATG | CAGCCTGTCATCCTCAATATC |
| ACAT1 | GCAGCTGTGCTGAGAATAC | CTTCCCATGCTGCTTTACTT |
| ACLY | CACTCCTCTGCTCGATTATG | GACTCCGATGAGACCATCTA |
| ACOX1 | CAACCATCGTATTCCCAGAG | CCGTAAGTCAGCTTGTTACTC |
| ACSL4 | GTGAAAGAATACCTGGACTGG | GACTGGTCAGAGAGTGTAA |
| ATGL | AACACCAGCATCCAGTTC | GTATCCCTGCTTGCACAT |
| CD36 | GGCTGTGTTTGGAGGTATT | GTACCTTCTTCGAGGACAAC |
| CPT1A | GACCGGGAGGAAATCAAAC | CTGGGATCCGGGAAGTATTA |
| CYP27A1 | TGCGCCAGGCTCTGAAC**G**AG | TCCACTTGGGGAGGAAGGTG |
| ELOVL1 | CTTCCATCACTCTGTGCTTC | GACATGCACGGAAGAGTTTA |
| ELOVL5 | ACCGCAGGAGAATCAGATA | CTTGCGCAGGATGAAGAA |
| FADS2 | CTTTGTCCTTGCTACCTCTC | GTGGTTCCACTTGGGTTT |
| FASN | TACGGCCCTCATTTCCA | CATGAAGCTCACCCAGTTATC |
| G0S2 | GATGGTGAAGCTGTACGTG | TGCACACAGTCTCCATCA |
| GAPDH | CCCACTCCTCCACCTTTGAC | TGTTGCTGTAGCCAAATTCGTT |
| GLS1 | TCCTCAACTGGCCAAATTC | CAGAAGGGAACTTTGGTATCTC |
| GPX4 | ATACGCTGAGTGTGGTTTG | GGCGAACTCTTTGATCTCTT |
| GSS | GCCATAGAGAATGAGCTACTG | AAACAGCCTTCGGTCTTG |
| HADHA | CAGAACTGCTGACACAGATG | CTTCACACCCTCCTGATAGA |
| HADHB | AAGACTCCTGCTCACACT | CTGGCCAGAAGCAATCAA |
| HIF-1α | AGCCGAGGAAGAACTATGA | CACACTGAGGTTGGTTACTG |
| HK1 | CAACAGCCACAGTCAAGAT | AAGGAAGACCCACCAAGA |
| HK2 | GAGTGGAGATGCACAACAA | CTGGACAATGTGGTCAAAGA |
| HMGCR | GTCATTCCAGCCAAGGTTGT | GGGACCACTTGCTTCCATTA |
| IDI | TCTGCTACAGCAAAGATCAG | CTCGGCTGGATTGCTTAAT |
| LDHA | GCCGTGATAATGACCAGCTT | TGGCAGCCTTTTCCTTAGAA |
| LDL-R | GAGGTGGCCAGCAATAGAATCT | GCCGTGGGCTCTGTCAAG |
| LIPE | CGTCAGGCTCATCTCCTAT | TTGGCCGTTGGACTTTATC |
| MAGL | GTGGATTCCATGCAGAAAGA | AAGAACCAGAGGCGAAATG |
| MYC | CGGATTCTCTGCTCTCCT | CTTCCTCATCTTCTTGTTCCTC |
| NR5A1 | GGAGTTTGTCTGCCTCAAGTTCA | CGTCTTTCACCAGGATGTGGTT |
| NR5A2 | TACCGACAAGTGGTACATGGAA | CGGCTTGTGATGCTATTATGGA |
| PFKL | CTCATCTACGAGGGCTATGA | GCTGGATGATGTTGGAGAC |
| PLIN1 | GAATTGGAGACTGAGGAGAAC | GTCTTCTGCAGGGTATGTG |
| PLIN2 | TGAGGAGAGACTGCCTATT | AGTAGTCGTCACAGCATCT |
| PPARα | TCTCAGGAAAGGCCAGTAA | AGCGTCTTCTCAGCCATA |
| SCARB1 | GGGCTCTTCACGGTGTTCAC | ACATTTGCCCAGAAGTTCCATT |
| SCD1 | CCCTGTATGGGATCACTTTG | GATGAGCTCCTGCTGTTATG |
| SLC1A5 | TGCCTTTGGGACCTCTT | TTGGCCACGCCATTATTC |
| SLC27A2 | GATATTGCGTCAGAGTTCCC | CTTCTCTGTCTGAGCCTTTG |
| SLC27A3 | CCCAGAGCATAACAGACAC | TGGCATTGCAGGATCTTC |
| SLC27A4 | GGTGCACAGCAGGTATTA | CCGATTCCCACGATGTTT |
| SLC2A1 | GCATGTGCTTCCAGTATGT | GTCTCAGGAACTTTGAAGTAGG |
| SLC6A9 | TCACCATGGCTTCCTACA | CCGAGGATGGAGAAGATGA |
| SLC7A11 | CGACCATTAATGCTGAGGAG | AGGAGAGGGCAACAAAGA |
| SQLE | GCTTCCTTCCTCCTTCATC | GTCATTCCTCCACCAGTAAG |
| SREBP1 | CGGAACCATCTTGGCAACA | GCCGGTTGATAGGCAGCTT |
| SREBP2 | GAAGCCCTCTATTGGATGATG | AGGTGAGGACACACAGAA |
