# Supplementary material for: A targetable antioxidant defense mechanism to EZH2 inhibitors enhances tumor cell vulnerability to ferroptosis
Source: Cell Death Dis. 2025 Apr 14;16(1):291. doi: 10.1038/s41419-025-07607-y (PMC11997205; doi:10.1038/s41419-025-07607-y)
Supplement: Supplementary file 5 — Original Data [file 41419_2025_7607_MOESM5_ESM.pptx]

## Slide 1
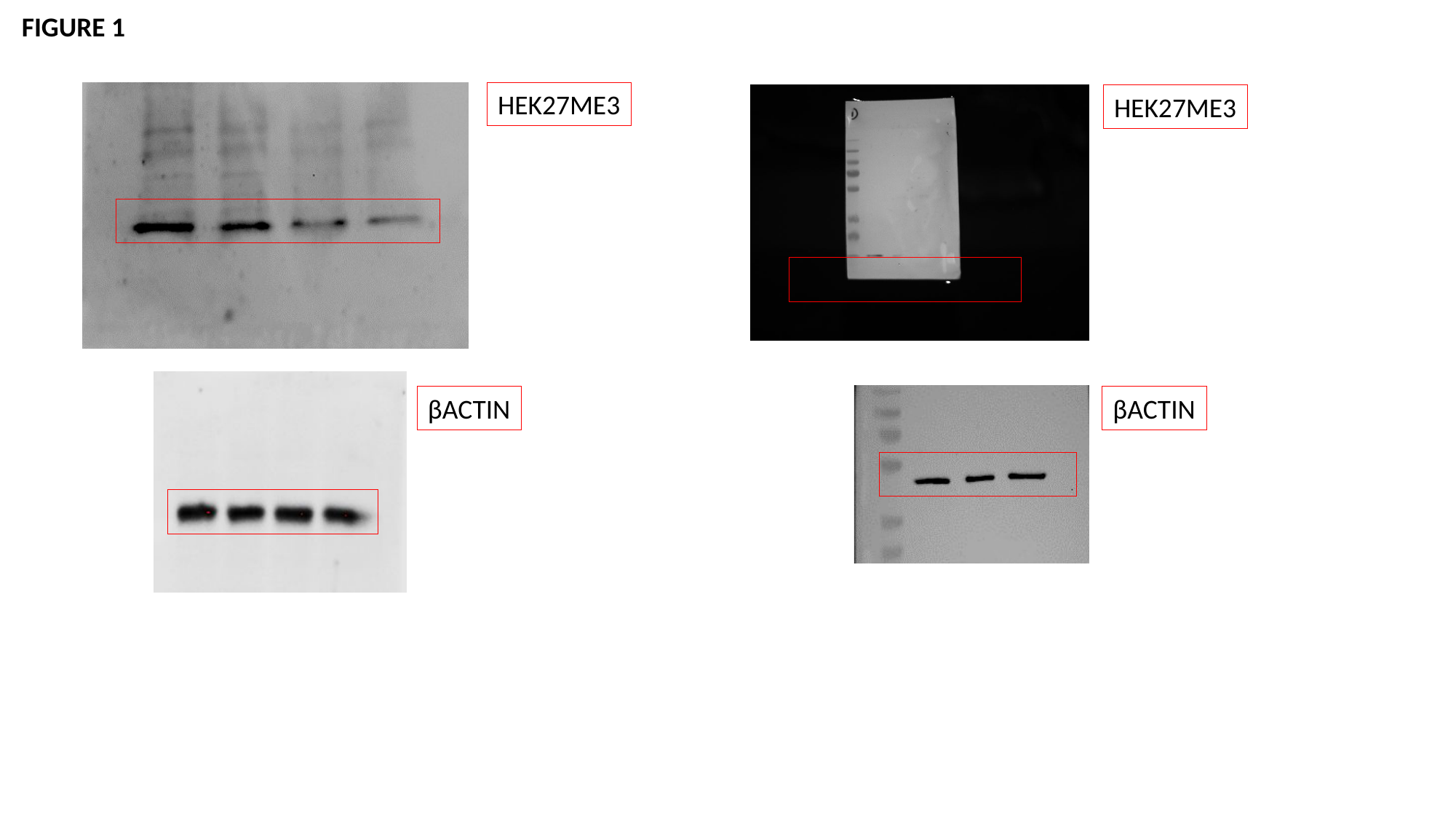

FIGURE 1
HEK27ME3
HEK27ME3
βACTIN
βACTIN

## Slide 2
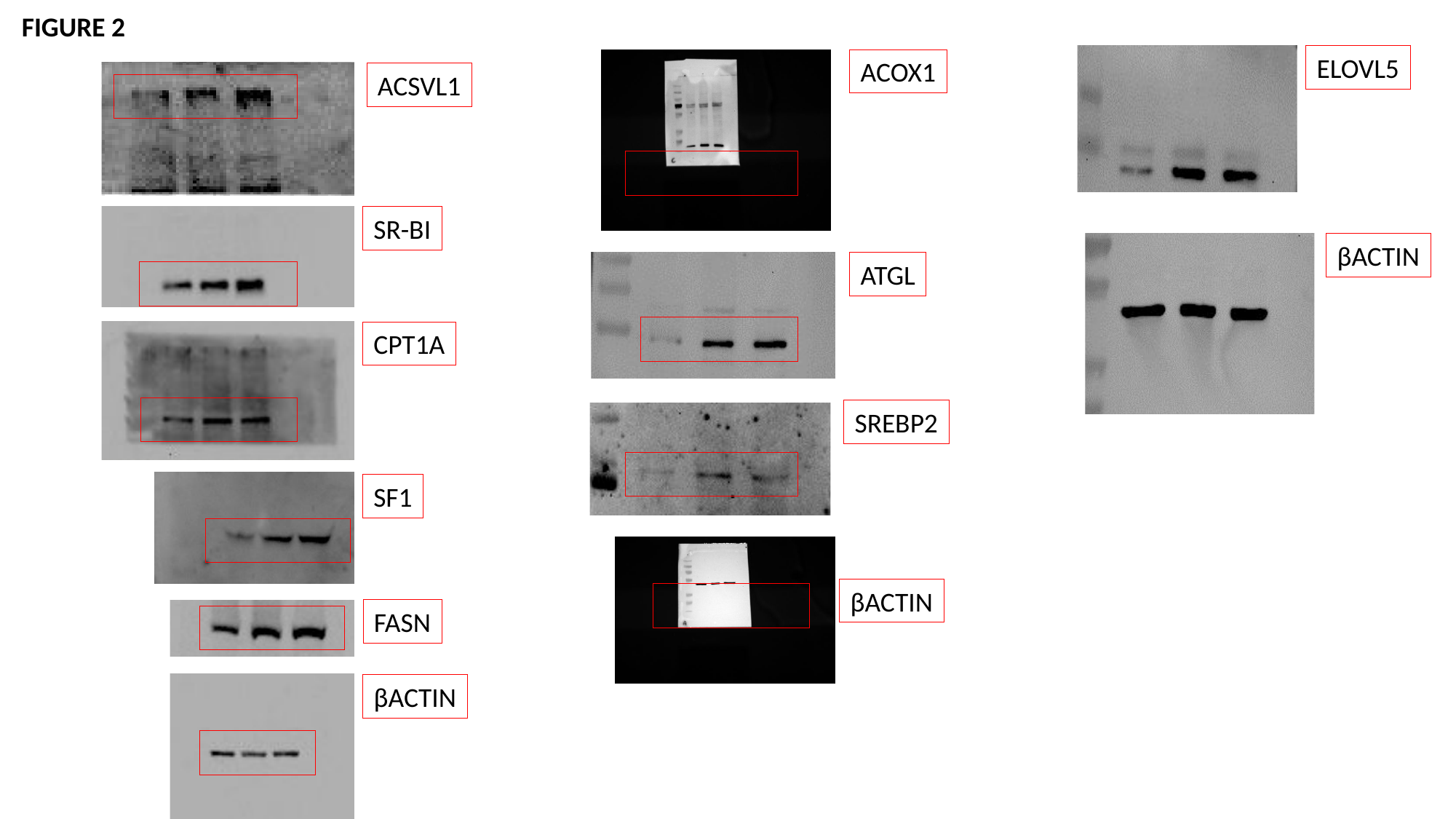

FIGURE 2
ELOVL5
ACOX1
ACSVL1
SR-BI
βACTIN
ATGL
CPT1A
SREBP2
SF1
βACTIN
FASN
βACTIN

## Slide 3
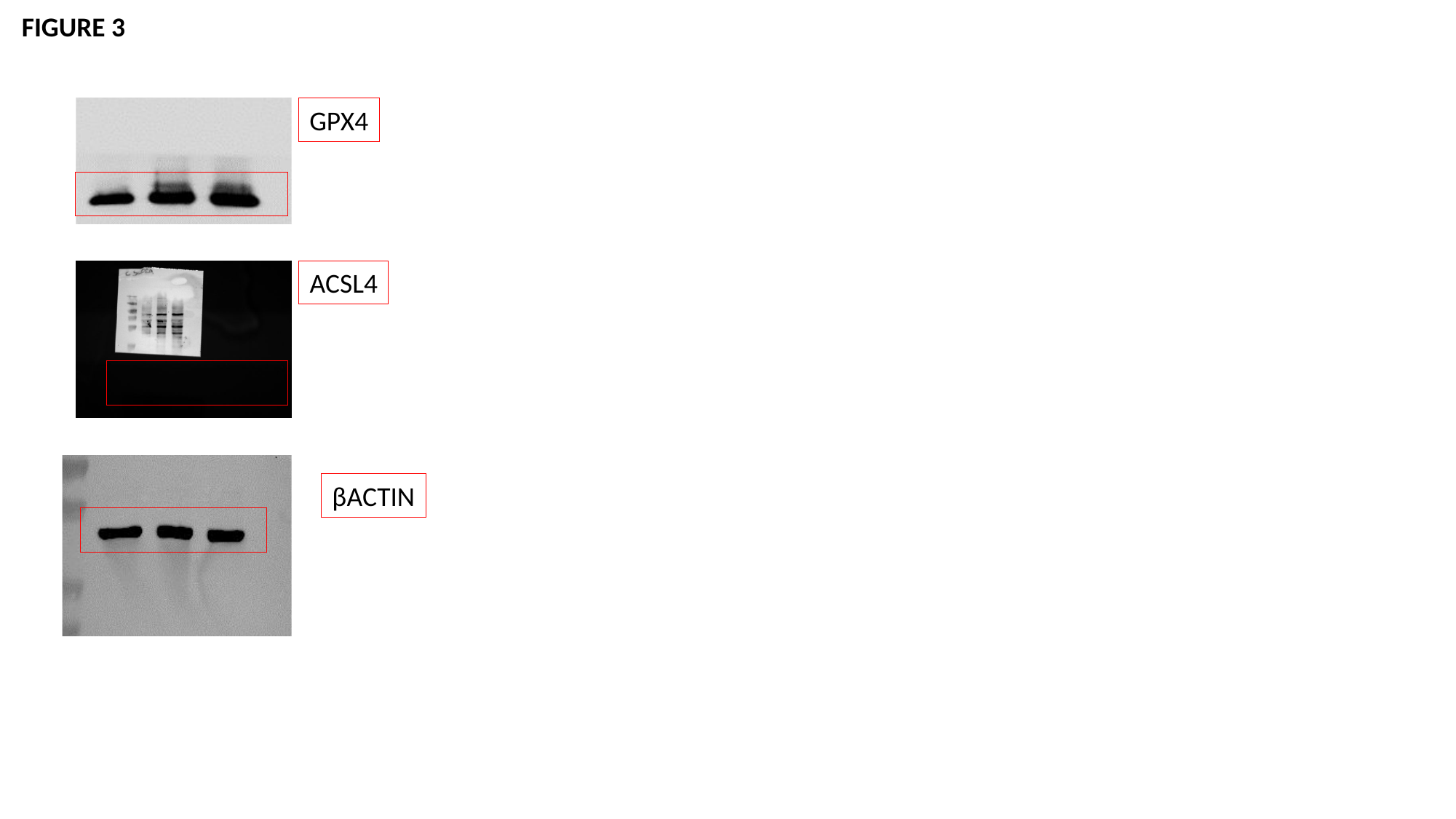

FIGURE 3
GPX4
ACSL4
βACTIN

## Slide 4
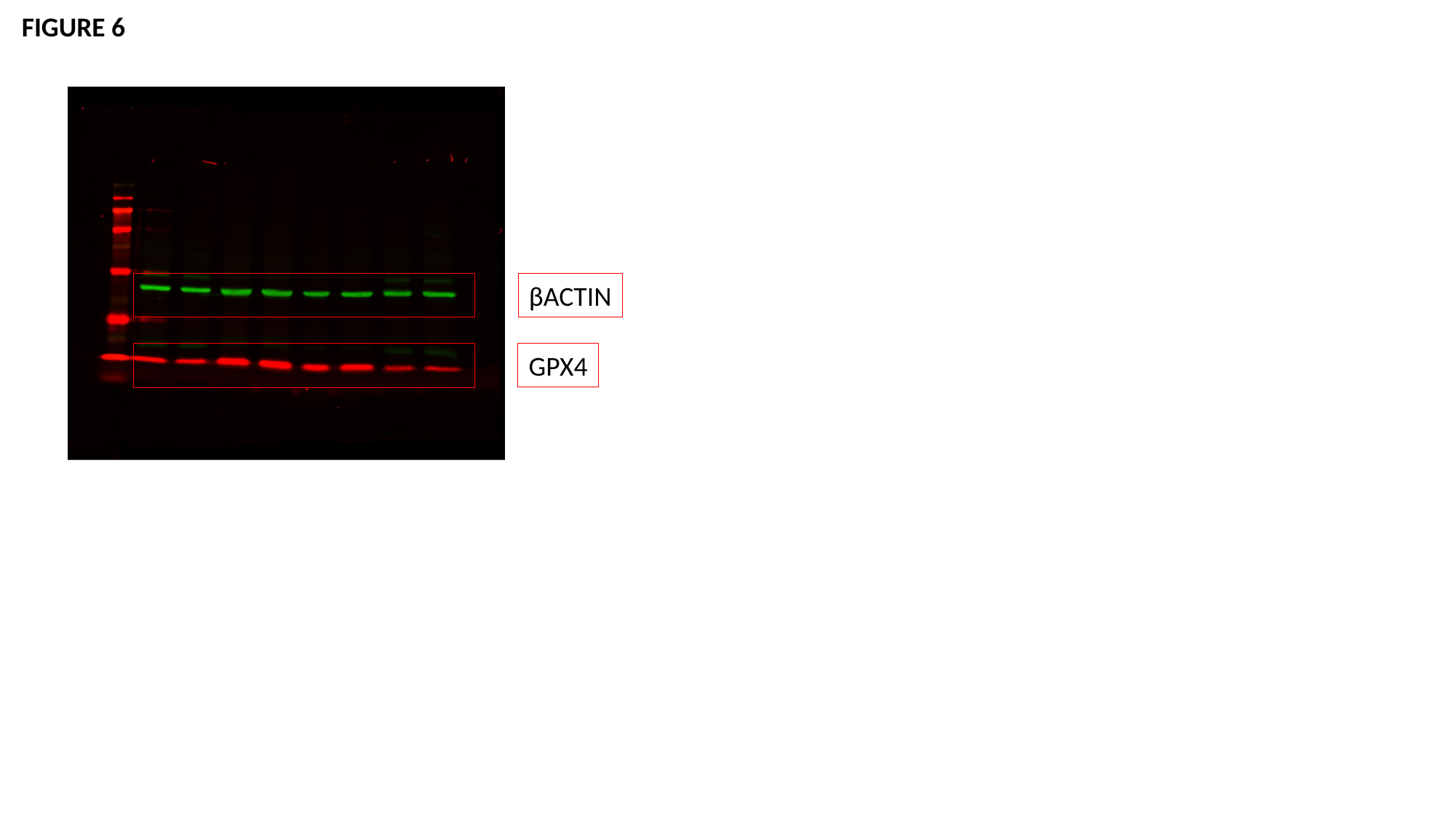

FIGURE 6
βACTIN
GPX4
